# Total Sedentary Time and Cognitive Function in Middle-Aged and Older Adults: A Systematic Review and Meta-analysis

**DOI:** 10.1186/s40798-022-00507-x

**Published:** 2022-10-12

**Authors:** Kirsten Dillon, Anisa Morava, Harry Prapavessis, Lily Grigsby-Duffy, Adam Novic, Paul A. Gardiner

**Affiliations:** 1 Faculty of Health Sciences, The University of Western Ontario, Kinesiology, London, ON Canada; 2 The University of Queensland, Brisbane, Australia; 3 Global Obesity Centre (GLOBE), Institute for Health Transformation, Deakin University, Geelong, VIC 3220 Australia; 4 School of Applied Psychology, Griffith University, Brisbane, Australia; 5 Faculty of Health, Engineering and Sciences, The University of Southern Queensland, Brisbane, Australia

**Keywords:** Systematic review, Meta-analysis, Middle-aged, Older adults, Sedentary behaviour, Cognition, Cognitive decline

## Abstract

**Background:**

An estimated 47 million people have dementia globally, and around 10 million new cases are diagnosed each year. Many lifestyle factors have been linked to cognitive impairment; one emerging modifiable lifestyle factor is sedentary time.

**Objective:**

To conduct a systematic review and meta-analysis of peer-reviewed literature examining the association between total sedentary time with cognitive function in middle-aged and older adults under the moderating conditions of (a) type of sedentary time measurement; (b) the cognitive domain being assessed; (c) looking at sedentary time using categorical variables (i.e., high versus low sedentary time); and (d) the pattern of sedentary time accumulation (e.g., longer versus shorter bouts). We also aimed to examine the prevalence of sedentary time in healthy versus cognitively impaired populations and to explore how experimental studies reducing or breaking up sedentary time affect cognitive function. Lastly, we aimed to conduct a quantitative pooled analysis of all individual studies through meta-analysis procedures to derive conclusions about these relationships.

**Methods:**

Eight electronic databases (EMBASE; Web of Science; PsycINFO; CINAHL; SciELO; SPORTDiscus; PubMed; and Scopus) were searched from inception to February 2021. Our search included terms related to the exposure (i.e., sedentary time), the population (i.e., middle-aged and older adults), and the outcome of interest (i.e., cognitive function). PICOS framework used middle-aged and older adults where there was an intervention or exposure of any sedentary time compared to any or no comparison, where cognitive function and/or cognitive impairment was measured, and all types of quantitative, empirical, observational data published in any year were included that were published in English. Risk of bias was assessed using *QualSyst.*

**Results:**

Fifty-three studies including 83,137 participants met the inclusion criteria of which 23 studies had appropriate data for inclusion in the main meta-analysis. The overall meta-analysis suggested that total sedentary time has no association with cognitive function (*r* = −0.012 [95% CI − 0.035, 0.011], *p* = 0.296) with marked heterogeneity (*I*^2^ = 89%). Subgroup analyses demonstrated a significant negative association for studies using a device to capture sedentary time *r* = −0.035 [95% CI − 0.063, − 0.008], *p* = 0.012). Specifically, the domains of global cognitive function (*r* = −0.061 [95% CI − 0.100, − 0.022], *p* = 0.002) and processing speed (*r* = −0.067, [95% CI − 0.103, − 0.030], *p* < 0.001). A significant positive association was found for studies using self-report (*r* = 0.037 [95% CI − 0.019, 0.054], *p* < 0.001). Specifically, the domain of processing speed showed a significant positive association (*r* = 0.057 [95% CI 0.045, 0.069], *p* < 0.001). For prevalence, populations diagnosed with cognitive impairment spent significantly more time sedentary compared to populations with no known cognitive impairments (standard difference in mean = −0.219 [95% CI − 0.310, − 0.128], *p* < 0.001).

**Conclusions:**

The association of total sedentary time with cognitive function is weak and varies based on measurement of sedentary time and domain being assessed. Future research is needed to better categorize domains of sedentary behaviour with both a validated self-report and device-based measure in order to improve the strength of this relationship. PROSPERO registration number: CRD42018082384.

**Supplementary Information:**

The online version contains supplementary material available at 10.1186/s40798-022-00507-x.

## Key Points

1. The association of total sedentary time with cognitive function varies based on the method of sedentary time measurement and the cognitive domain being assessed
2. Populations that have been diagnosed with mild cognitive impairment or dementia spend significantly more time sedentary compared to cognitively healthy populations.
3. Future research is needed to investigate associations of individual sedentary behaviours with cognitive function and examine the impact of cognitive load on this association.

## Introduction

Cognitive function refers to multiple mental abilities including learning, thinking, reasoning, remembering, problem solving, decision making and attention [1]. Dementia can be defined simply as a significant loss of cognitive function that impacts functions of daily living [2]. An estimated 47 million people have dementia globally and around 10 million new cases are diagnosed each year [3]. Unfortunately, this number will continue to rise at an exponential rate due to a global increase in the number of people living to older age [4]. Dementia has a major impact on the individual, but also has detrimental physical, emotional, psychological, social, and economic effects on caregivers, families, and society as a whole [5]. It is estimated that the total global societal cost of dementia is US$818 billion per year (1.1% of global gross domestic product); making it a public health priority [6, 7].

With the absence of a pharmacological treatment for the disease, current medications can only modestly alleviate symptoms [8]. Thus, other strategies (i.e., lifestyle modifications) are imperative for addressing the forthcoming dementia pandemic. Although cognitive decline tends to materialize later on in life, it is experienced by every individual at different severities and rates [1]. The latest Lancet review published in 2020 on dementia prevention, intervention and care concluded that 40% of worldwide dementia cases can be attributed to 12 modifiable risk factors [9], which is three more risk factors than the original review published in 2017 [10]. While we know a lot about some risk factors, there may be other unexplored factors, for example, targeting the reduction of sedentary time and how it may impact cognitive function. Despite the well-known benefits of moderate to vigorous physical activity, only 15% of adults aged 65–79 achieve at least 150 min of moderate to vigorous physical activity per week [11] and in addition, spend an average of 10.1 h/day being sedentary [12].

The associations between sedentary time and non-communicable diseases such as cardiovascular disease, type 2 diabetes, some forms of cancers, as well as all-cause mortality are now well established [13]. Sedentary behaviour is defined as any waking behaviour in a seated or lying posture while expending less than or equal to 1.5 metabolic equivalents of energy [14]. The evidence on the health risks associated with too much sitting is now informing public health guidelines. For example the Canadian 24-h movement guidelines recommend that in addition to accumulating 150 min of moderate to vigorous physical activity per week, people should keep prolonged sitting time to a minimum, advising no more than eight hours of sitting per day [15].

Sedentary time has become a known modifiable determinant of health and an important predictor of healthy aging [16–19]. More recently, studies have emerged suggesting that higher levels of sedentary time may also be linked to lower levels of cognitive function and an increased risk of cognitive decline [20–22]. Previous reviews have investigated the relationship between sedentary behaviour and cognitive function [23–26]. From these reviews, we know that the relationship is inconsistent, and observed relationships are rather weak. More specifically, Falck et al. (2016) suggested higher levels of sedentary behaviour were associated with lower cognitive performance [24]. This was concluded from eight observational studies including adults 40 years and over. A later review by Copeland and colleagues (2017) investigated the relationship in adults 60 years and older [23]. They were able to include 14 studies, including five studies featured in Falck’s 2016 review [24]. Of these, only half reported finding associations between increased sedentary behaviour and decreased cognitive function and the results were not differentiated according to the exposure and outcome measures used (i.e., self-report versus device or specific cognitive domains). The review by Loprinzi (2019) included humans of all ages and focused specifically on memory, including 25 studies [25]. Loprinzi observed a conflicting association of sedentary behaviour with cognitive function. Results from the most recent review are not much clearer. Olanrewaju et al. (2020) also found varied and inconclusive evidence on the relationship between the two variables [26]. The main difference of this review from the preceding ones was that it excluded studies involving participants diagnosed with dementia. Out of the 18 studies included, seven of these studies reported associations between increased sedentary behaviour time and decreased cognitive function. Only four of the 18 studies were consistent with those included in the Copeland (2017) review [23]. Despite including 18 studies, there were no interventions identified; and there was too much heterogeneity to perform a meta-analysis. The current systematic review builds on these previous ones, addressing specific conceptual and methodological issues that these reviews could not avoid due to the nature of the literature at that time.

Although the definition of sedentary behaviour was properly defined in previous reviews, the large-scale heterogeneity found within the available studies at the time could stem from the broadly defined study exposure (i.e., sedentary behaviour) needed in order to synthesize the current state of the literature. In other words, a specific domain of sedentary behaviour (i.e., television viewing) needed to be synthesized alongside studies measuring total sedentary time. This is problematic as we know that television viewing is a poor proxy of overall sedentary time [27]. Thus, no reviews have specifically aimed to review the association of total sedentary time with cognitive function. Secondly, the inconsistent and weak relationships between sedentary behaviour and cognitive function reported in previous reviews suggests that this relationship needs to be examined under certain moderator conditions. None of the reviews examined the association between studies that have used self-reported measures versus those that have used a device (i.e., activPAL™). This is an important moderator to investigate as self-report has been shown to underestimate sedentary time when compared to device-based measurements [23]. Although touched upon in the review by Falck (2016), another moderator that warrants attention is which specific domains of cognition are most affected by total sedentary time. Insight here will assist researchers’ focus on cognitions that are more salient to total sitting time. Third, no previous reviews have interpreted the findings based on a threshold or cut-off that compares two categorical groups (i.e., high versus low sedentary time) as opposed to comparing the relationship using continuous variables. It is unknown whether this relationship will be more concrete if more extreme groups or categorical groups are used; this may have implications for how researchers collect and analyze total sitting time data. Fourth, no previous review has interpreted the associations based on how sedentary time was accumulated throughout the day (i.e., short, frequent bouts vs. longer uninterrupted bouts). Recent investigations have highlighted the negative aspects of accumulating prolonged bouts of sedentary time regardless of meeting physical activity guidelines [17]. Thus, it is recommended to incorporate short bouts of activity to break up sedentary periods throughout the day [28]. Fifth, no previous reviews have synthesized the findings based on the prevalence of sedentary time in populations diagnosed with mild cognitive impairment or dementia versus populations deemed as cognitively healthy. Since the older adult population is the most sedentary and inactive population, it makes it hard to tease out a bidirectional relationship (i.e., is more sitting causing cognitive decline or is cognitive decline resulting in more sitting?). Therefore, differentiating populations that may be more susceptible to increased amounts of sitting, (i.e., people with cognitive impairment, transitioning into retirement, etc.) could help tease out any mixed association. This also highlights the importance of including both middle-aged and older adults. Sixth, experimental studies were scarce at the time of these previous reviews, but with the growing interest in this relationship, synthesizing any experimental studies aiming to reduce or break up sedentary time is important as it could illustrate whether we are able to manipulate cognitive function with sitting time. Seventh and finally, due to the heterogeneity of the literature at the time of these previous reviews, they were not able to perform a meta-analysis to quantify the effect size of the relationship. Since these reviews, there have been many more studies published on the relationship of sedentary behaviour with cognitive function, which allows this review to have a narrower view of the exposure variable, sedentary time, alongside being able to evaluate the relationship with the aforementioned moderators.

Addressing the abovementioned issues, the overarching objective of this study was to conduct a systematic review to explore the relationship of total sedentary behaviour time with cognitive function in middle-aged and older adults. A specific objective was to examine the relationship under the following moderator conditions: a) self-reported versus device based sedentary time measurement; b) the cognitive domain being assessed (e.g., working memory, processing speed, etc.); c) looking at sedentary time using categorical variables (i.e., high versus low sedentary time) and d) the pattern of sedentary time accumulation (e.g., longer versus shorter bouts). We also aimed to examine the prevalence of sedentary time in healthy versus cognitively impaired populations. Additionally, we aimed to explore how experimental studies reducing or breaking up sedentary time affects cognitive function. Lastly, we aimed to conduct a quantitative pooled analysis of all individual studies through meta-analysis procedures to derive conclusions about these relationships. In doing so, we intended to determine whether the aforementioned variables on their own, or in conjunction with one another served to change the strength and/or direction of relationships between total sedentary time and cognitive function.

## Methods

The protocol for this review is registered on PROSPERO (registration number: CRD42018082384). The review and meta-analysis were also completed in accordance with the Preferred Reporting Items for Systematic Reviews and Meta-Analyses guidelines (PRISMA). This paper was also conducted in accordance with the PRISMA in Exercise, Rehabilitation, Sports Medicine, and Sports Science guidelines (PERSIST).

### Search Strategy

We searched the following electronic databases from inception to February 13th, 2021: Excerpta Medica (EMBASE); Web of Science; PsycINFO; Cumulative Index to Nursing and Allied Health Literature (CINAHL); Scientific Electronic Library Online (SciELO); SPORTDiscus; PubMed; and Scopus. The databases were searched using a combination of controlled vocabulary (MeSH) and free text terms. The review authors along with librarians at The University of Queensland and Western University developed the search strategy. The search included terms related to the exposure (i.e., sedentary behaviour), the population (i.e., middle-aged and older adults), and the outcome of interest (i.e., cognitive function) (See Additional file 1 for an example search strategy for Scopus). All database searches were appropriately revised to suit the specific database. Where the database software had the function, we used "forward" citation searches (showing the sources that cite these articles) on the studies we included in this systematic review. Additionally, reference lists of all relevant reviews on cognitive outcomes and sedentary behaviour from the last 10 years were hand-searched. These reviews were identified in the initial title and abstract screening and through a search of our key terms in Cochrane. All additional articles identified through these other sources were subject to the same eligibility criteria and screening process as those found through the electronic database searches.

### Selection Criteria

#### Types of Participants

The focus of this review was middle-aged and older adults. Therefore, we included studies where the mean age of the population was aged 40 or over [29]. Studies from any country were included. If the data set had been used more than once, the publication most relevant and appropriate for the current review was included.

#### Types of Interventions/Exposure

This review examined studies where the intervention or exposure was sedentary behaviour, as defined by each individual study. The definition of sedentary behaviour is sitting or lying behaviours during waking time that expend low levels of energy (≤ 1.5 metabolic equivalents) [14]. Studies were included if they reported the total time spent ‘sedentary’ per day or week, or if they reported the percentage of daily waking hours in ‘sedentary behaviour’ per day or week. Thus, any reference to ‘sedentary time’ in the current review can be interpreted as ‘total’ or all-encompassing sedentary time unless stated otherwise. Studies were excluded if they defined and/or measured sedentary behaviour as a lack of physical activity, included sleep time in the reported sedentary behaviour time, only measured leisure sedentary time, or only measured a specific domain of sedentary behaviour. Studies were also excluded if they were a multicomponent lifestyle intervention where sedentary behaviour was only one component of the intervention. In other words, if sedentary behaviour was only a ‘control’ condition of the intervention, it was excluded. Studies specifically investigating bedrest or designed for cognitive rehabilitation or training were excluded.

#### Types of Comparison

Studies with any comparators or no comparators were eligible for inclusion.

#### Types of Outcome Measures

Studies measuring cognitive function and/or cognitive impairment/decline (i.e., dementia or mild cognitive impairment [MCI]) using a recognised method or measure were included. Cognitive outcomes from each study were tabulated and categorized based on the authors’ classification of which domain the task measured (i.e., processing speed, episodic memory, etc.). If the task was used in more than one study and the authors reported different domains, discrepancies were solved by the current study’s authors using guidance from an article which reviewed the general structures of cognitive domains, along with assessment strategies for differentiating them [30]. The various outcomes of cognitive function from each study were grouped into one or more of the following domains: (1) processing speed; (2) episodic memory; (3) global cognitive function; (4) motor skills and construction; (5) executive function; (6) cognitive flexibility and (7) working memory. The definition and acceptable cognitive tests for each domain are described in Additional file 2. Studies that measured cognitive function as defined by brain volume or cerebral blood flow using Magnetic Resonance Imaging (MRI) or Positron Emission Tomography (PET) were excluded.

#### Types of Studies

Quantitative empirical studies published in any year were included, including observational (cross-sectional and/or longitudinal) and intervention/experimental studies. Discussion articles, conference proceedings, book chapters, theses or commentaries not presenting empirical research in a peer-reviewed journal were excluded. Studies published in a language other than English were also excluded.

#### Selection of Studies

Titles and abstracts of the identified studies were independently checked by two review authors using Covidence systematic review software and those that clearly did not meet the inclusion criteria were excluded. Full texts were then also reviewed by two authors. At both stages of the screening process, the authors discussed any discrepancies in their initial judgements and reached a consensus.

### Data Extraction

Data from each of the included studies were extracted independently by one review author and checked by a second author for accuracy. The following data from each of the included studies were extracted and can be found in Tables 1, 2, 3 and 4: (1) author name and year; (2) study design; (3) participant characteristics (country, number of participants in each group, mean age, percent of female participants, and sedentary time reported; (4) measure of exposure (i.e., sedentary behaviour); (5) measure of outcome (i.e., cognitive function); (6) covariates used in the least and fully adjusted models (if applicable) and (7) main findings/numerical results for each outcome of interest (i.e., correlation coefficients, mean [SD], effect sizes, p values, etc.). Additional information that was extracted, but not reported within Tables 1, 2, 3 and 4 consisted of: (1) study objectives; (2) source of recruitment/method; (3) inclusion/exclusion criteria; (4) number of groups and method of group allocation (if application); (5) type of data (i.e., binary or continuous) for both exposure and outcome variables; (6) time frame of intervention/observation; (7) statistical methodology (i.e., subgroup analysis, intention to treat, etc.); (8) conclusion; (9) limitations identified by the authors and 10) any declared conflict of interest. If any study reports were incomplete or if data were missing, corresponding study authors were contacted via email.

**Table 1:** Summary and characteristics of cross-sectional and longitudinal studies reporting an association of sedentary behaviour with cognitive function

Authors (year) Country Study design Pinwheel number	Participants Mean age (Mage) % F (female) SB time (min)	Device or self-report (measure of sedentary behaviour)	Domain (outcome measure)	Covariates adjusted for	Results
Amagasta et al. (2020) [77] *Japan CS Prevalence* *Study number in pinwheel* = *1*	*Cognitive decline* *n* = 48, Mage = 77.6 (5.4), %*F* = 52, SB time = 476 *Non-cognitive decline* *n* = 463, Mage = 73.0 (5.4), %*F* = 53, SB time = 442 Population: mixed	**Device** (Active style Pro HJA-750C)	**Global Cognitive Function** (MMSE) ≤ 23 = Cognitive Function Decline	Model 1: unadjusted Model 4: Gender, age, education, BMI, living arrangements, working status, smoking, alcohol use, past history of stroke, medication for hypertension, dyslipidemia, diabetes	**SB relative to other behaviours**: OR 1.06; 95% CI [0.42, 2.72] **SB and cognitive function:** *Model 1:* OR 1.30 [0.63, 2.70] *Model 4:* OR 0.96 [0.38, 2.39]
Bollaert et al. (2019) [68] *USA CS Prevalence Pattern* *Study number in pinwheel* = *39*	*Healthy group* *n* = 40, Mage = 66.5 (6.7), %*F* = 63, SB time = 534 *Multiple Sclerosis group* *n* = 40, Mage = 65.3 (4.3), %*F* = 63, SB time = 540 Population: mixed	**Device** (Actigraph)	**Processing Speed** (PASAT) **Episodic Memory** (CVLT-II)	Not stated	***Total SB*** ***Healthy Controls (r)*** SDMT: − 0.38 CVLT-II: − 0.13 BVMT-R: − 0.03 PASAT: − 0.11 ***MS Group (r)*** SDMT: − 0.15 CVLT-II: 0.20 BVMT-R: − 0.08 PASAT: 0.08
Bojsen-Moller et al. (2019) [50] *Sweden CS* *Study number in pinwheel* = *2*	*n* = 334, Mage = 42.4 (9.1), %*F* = 68, SB time = 565 Population: non-clinical	**Device** (Actigraph GT3X)	**Processing Speed** (TMT A, Digit Symbol) **Working Memory** (DSB, N-back (2-back), AOS, TMT B) **Episodic Memory** (Free Recall, Word Recognition) **Executive Function** (Stroop) **Cognitive Flexibility** (TMT B)	Model 2: age, gender, education, % of daytime in sedentary behaviour Model 4: age, gender, education, % of daytime in sedentary behaviour and VO2max	**SB and cognitive outcomes: *β*, [95% CI]** *Model 2* *Digit symbol:* 0.004 [− 0.145, 0.138] *Free recall:* 0.125 [− 0.011, 0.266], *p* < 0.05 *Digit span backwards:* 0.003 [− 0.139, 0.146] *2-back (accuracy):* − 0.022 [− 0.165, 0.120] *2-back (reaction time):* − 0.053 [− 0.195, 0.087] *Word recognition:* 0.035 [− 0.107, 0.177] *AOS (recalled sets):* − 0.011 [− 0.159, 0.136] *AOS (accuracy)*: − 0.022 [− 0.165, 0.120] *Stroop:* 0.012 [− 0.131, 0.155] *TMT A:* 0.042 [− 0.103, 0.190] *TMT* B: − 0.032 [− 0.194, 0.126] *Model 4* *Digit symbol: − *0.047 [− 0.219, 0.126] *Free recall:* 0.136 [− 0.003, 0.280], *p* < 0.05 *Digit span backwards: − *0.003 [− 0.149, 0.142] *2-back (accuracy):* 0.060 [− 0.085, 0.207] *2-back (reaction time): − *0.052 [− 0.198, 0.092] *Word recognition:* 0.071 [− 0.071, 0.215] *AOS (recalled sets):* 0.010 [− 0.140, 0.161] *AOS (accuracy): − *0.006 [− 0.152, 0.140] *Stroop: − *0.016 [− 0.162, 0.129] *TMT A:* 0.04 [− 0.108, 0.191] *TMT* B: − 0.044 [− 0.210, 0.118]
Burzynska et al. (2020) [69] *USA CS* *Study number in pinwheel* = *40*	*n* = 228, Mage = 65.3 (4.5) %*F* = 68, SB time = 537 Population: non-clinical	**Device** (ActiGraph)	**Processing Speed** (DS, Digit Symbol, Pattern comparison, letter comparison) **Episodic Memory** (Word recall, Logical memory, Paired associates)	Model 1: age, sex, education, adult education, light, and moderate to vigorous PA	β (SE) **Processing speed:** − 0.009 (0.001) *p* = 0.900 **Episodic Memory:** β = 0.088 (0.001) *p* = 0.245
Cukic et al. (2018) [35] *Scotland* CS/LO *Pattern* *Study number in pinwheel* = *3*^a,b,c^	LBC1936 cohort *n* = 271, Mage = 79.0 (0.4) % *F* = 48, SB time = 627 Population: non-clinical 3^a^	**Device** (activPal3)	**Global Cognitive Function** (general cognitive ability factor (g) computed from 6 tests taken from the ﻿WAIS (﻿Matrix Reasoning, Block Design, Letter-Number Sequencing, Symbol Search, DSB, and Digit Symbol), Moray Houst Test No. 12 (MHT), Alice Heim 4 test (AH4)) **Processing Speed** (Four-choice RT) **Motor Skills and Construction** (Simple RT)	Model 1: age and sex Model 3: age, sex, education, long standing illness	**Cog ability & total SB: *β*, [95% CI]** *Model 1* *g-wave 4:* − 0.06 [− 0.18, 0.06], *p* = 0.36 *Simple RT:* 0.03 [− 0.09, 0.15], *p* = 0.61 *Choice RT: − *0.10 [− 0.22, 0.02], *p* = 0.09 *MHT change age 11–79: − *0.09 [− 0.21, 0.03], *p* = 13 *Model 3* *g-factor: − *0.01 [− 0.15, 0.13], *p* = 0.9 *Simple RT:* 0.02 [− 0.1, 0.14], *p* = 0.77 *Choice RT: − *0.12 [− 0.24, 1.0], *p* = 0.05 *MHT change age 11–79*: − 0.06 [− 0.20, 0.08], *p* = 0.34
	Twenty-07 1950’s cohort *n* = 310, Mage = 64.6 (0.9), %*F* = 53.2, SB time (%) = 60.8 Population: non-clinical 3^b^			Model 1: age and sex Model 4: age, sex, education, long standing illness, employment status	**Cog ability & total SB: *β*, [95% CI]** *Model 1* *AH4 wave 5:* − 0.08 [− 0.04, 0.20], *p* = 0.18 *Simple RT wave 5*: 0.12 [0.00, 0.24], **p = 0.04** *Choice RT wave 5:* 0.09 [− 0.03, 0.21], *p* = 0.13 *Model 4* *AH4 wave 5: − *0.06 [− 0.20, 0.08], *p* = 0.39 *Simple RT:* 0.06 [− 0.06, 0.18], *p* = 0.26 *Choice RT:* 0.05 [− 0.07, 0.17], *p* = 0.41
	Twenty-07 1930’s cohort *n* = 119, Mage = 83.4 (0.6), %*F* = 55, SB time (%) = 68 Population: non-clinical 3^c^			Model 1: age and sex Model 3: age, sex, education, long standing illness	**Cog ability & total SB: *β*, [95% CI]** Model 1 *AH4 wave 1:* − 0.08 [− 0.28, 0.12], *p* = 0.41 *AH4 wave 5:* − 0.07 [− 0.27, 0.13], *p* = 0.49 *Simple RT wave 5:* 0.04 [− 0.14, 0.22], *p* = 0.70 *Choice RT wave 5:* 0.10 [− 0.14, 0.34], *p* = 0.42 Model 3 *AH4 wave 1:* − 0.08 [− 0.30, 0.14], *p* = 0.45 *AH4 wave 5:* − 0.04 [− 0.26, 0.18], *p* = 0.75 *Simple RT wave 5:* 0.02 [− 0.18, 0.22], *p* = 0.82 *Choice RT wave 5:* 0.10 [− 0.15, 0.35], *p* = 0.42
Ehlers et al. (2018) [41] *USA* CS *Isotemporal* *Study number in pinwheel* = *5*	*n* = 271, Mage = 57.8 (9.5), %*F* = 100, SB time = 600 Population: clinical	**Device** (Actigraph) Isotemporal: Substituting 30 min of sedentary time with 30 min of light-intensity activity, MVPA, and sleep	﻿**Processing Speed** (TMT A) **Cognitive Flexibility** (TMT B, Task-switching) **Working Memory** (TMT B)	Single effect: age, months of hormonal therapy, chemotherapy, total time Partition effect: age, months of hormonal therapy, chemotherapy, total time, other behaviours	**Total SB time and cognitive function** ***B* 95% CI** *Single effect* *Task switch-switch:* 2.06 [− 7.07, 11.16] *Task switch-stay:* − 1.47 [− 9.62, 6.69] *TMT A*: − 0.97 [− 1.85, − 0.08], *p* < 0.05 *TMT B*: − 1.19 [− 2.41, 0.03] *Partition effect* *Task switch-switch: − *8.76 [− 22.26, 4.76] *Task switch-stay:* − 5.94 [− 18.07, 6.18] *TMT A*: − 0.75 [− 2.05, 0.55] *TMT B: − *0.92 [− 2.71, 0.87] Replacing 30 min of sedentary time with 30 min of MVPA yielded faster reaction times on Task-Switch stay (*B* = −29.37, *p* = 0.04) and switch (*B* = −39.49, *p* = 0.02) trials Replacing sedentary time with light-intensity activity was associated with slower Trails A (*B* = 1.55 *p* = 0.002) and Trails B (*B* = 1.69, *p* = 0.02) completion
Ekblom et al. (2019) [90] *Sweden* CS *Study number in pinwheel* = *6*	*n* = 216, age range = 54–66, %*F* = 56, SB time = 461 Population: non-clinical	**Device** (ActiGraph)	**Episodic Memory** (Verbal Memory) **Motor Skills and Construction** (Rey Complex Figure) **Processing Speed** (SDMT, TMT A, Stroop 1) **Cognitive Flexibility & Working Memory** (TMT B) **Executive Function** (Stroop 2 & 3)	age, gender, smoking, education, born outside Sweden	**SB and cognitive function (*β*)** *verbal memory (verbatim):* 0.136, *p* < 0.05 *verbal memory (direct Syn):* 0.137, *p* < 0.05 *verbal memory (delayed Verbatim):* 0.119, *p* < 0.1 *verbal memory (delayed Syn):* 0.134, *p* < 0.1 *Stroop 1:* not significant *Stroop 2: − *0.141, *p* < 0.05 *Stroop 3: − *0.137, *p* < 0.01 *SDMT:* 0.126, *p* < 0.01 *TMT A:* not significant *TMT B: − *0.113, *p* < 0.01 *RCF 1:* 0.109, *p* = 0.139 *RCF 2:* 0.071, *p* = 0.342 *RCF 3: − *0.027, *p* = 0.717
English et al. (2016) [74] *Australia* *CS Pattern* *Study number in pinwheel* = *7*	*n* = 50, Mage = 67.2 (11.6), % *F* = 34, SB = nr Population: clinical	**Device** (activPAL)	**Global Cognitive Function** (MoCA)	waking hours	**MoCA with total sitting time** *r* = 0.153, *p* = 0.3
Ezeugwu et al. (2017) [70] *Canada CS Pattern* *Study number in pinwheel* = *8*	*n* = 30, Mage = 63.8 (12.3), %*F* = 43, SB time = 674 Population: clinical	**Device** (activPAL)	**Global Cognitive Function** (MoCA)	Not reported	**SB time and MoCA** *r* = −0.08, *p* > 0.05
Falck et al. (2017) [55] *Canada CS Prevalence Pattern* *Study number in pinwheel* = *10*	*Probable MCI* *n* = 81, Mage = 72.5 (7.6), %*F* = 60, SB time = 595 *Without MCI* *n* = 69, Mage = 69.4 (6.4), %*F* = 78, SB time = 542 Population: mixed	**Device** (MotionWatch8)	**Global Cognitive Function** (MoCA, ADAS-Cog Plus) Probable MCI = MoCA < 26	Age, sex, education	**SB and ADAS-Cog Plus (*β***) *% Sedentary time*: 0.007, p = 0.089 *Average 30* + *min bouts/day*: 0.061, *p* = 0.016 **SB and ADAS-Cog Plus Based on MCI Status (*β***) *non-MCI* % Sedentary time: 0.012, *p* = 0.038 Average 30 + min bouts/day: 0.075, *p* = 0.064 *MCI* % Sedentary time: < 0.001, *p* = 0.948 Average 30 + min bouts/day: 0.033, *p* = 0.282
Fanning et al. (2017) [42] *USA CS Isotemporal* *Study number in pinwheel* = *12*	*n* = 247, Mage = 65.4 (4.6), %*F* = 68, SB time = 534 Population: non-clinical	**Device** (Actigraph) Isotemporal: Substituting 30 min of sedentary behavior with 30 min of light activity, moderate-to-vigorous physical activity (MVPA), or sleep	**Working Memory** (computer-based task) **Cognitive Flexibility** (Task-switching)	Age, gender, race	**SB and Working memory** 2-item accuracy *r* = −0.106, *p* = 0.133 3-item accuracy *r* = −0.040, *p* = 0.575 4-item accuracy *r* = −0.081, *p* = 0.249 2-item reaction time *r* = 0.052, *p* = 0.460 3-item reaction time *r* = 0.013, *p* = 0.851 4-item reaction time *r* = 0.040, *p* = 0.566 ***SB and Cognitive Flexibility*** Single avg accuracy: *r* = −0.061, *p* = 0.391 Mixed repeat avg acc: *r* = 0.045, *p* = 0.532 Mixed switch avg acc: *r* = 0.006, *p* = 0.933 Global switch cost acc: *r* = −0.086, *p* = 0.228 Local switch cost acc: *r* = 0.084, *p* = 0.240 Single avg RT: *r* = 0.019, *p* = 0.794 Mixed repeat avg RT: *r* = 0.067, *p* = 0.352 Mixed switch avg RT: *r* = 0.070, *p* = 0.329 Global switch cost RT: *r* = 0.046, *p* = 0.525 Local switch cost RT: *r* = 0.026, *p* = 0.712 Substitution of sedentary time with MVPA was associated with higher accuracy on 2-item (*B* = .03, *p* = .01) and 3-item (*B* = .02, *p* = .04) working memory tasks, and with faster reaction times on single (*B* = −23.12, *p* = .03) and mixed-repeated task-switching blocks (*B* = −27.06, *p* = .04) Substitution of sedentary time with sleep was associated with marginally faster reaction time on mixed-repeated task-switching blocks (*B* = −12.20, *p* = .07) and faster reaction time on mixed-switch blocks (*B* = 17.21, *p* = .05), as well as reduced global reaction time switch cost (*B* = −16.86, *p* = .01)
García-Hermoso et al. (2018) [48] *Chile* *CS* *Study number in pinwheel* = *13*	*n* = 989, Mage = 74.1 (7.0), %*F* = 61, SB time = 225 Population: mixed	**Self-report** (Global Physical Activity Questionnaire)	**Global Cognitive Function** (modified-MMSE) ≤ 13 or less considered cognitively impaired	Model 1: age, sex, education level, BMI, social characteristics (living alone), alcohol and drug use, tobacco intake, depression Model 2: age, sex, BMI, social characteristics (education level and living alone), alcohol and drug use, tobacco intake, depression, MVPA	**Odds Ratio [95% CI]** 1.00, non-sedentary/active (ref group) 1.63 [0.92 to 4.75] non-sed/inactive, *p* = 0.084 1.91 [1.83 to 3.75] sed/active, *p* = 0.011 4.65 [2.50 to 6.31] sed/inactive, *p* < 0.001 **SB and cognition** *Model 1: β* = −0.063, *p* = < 0.001 *Model 2: β* = −0.046, *p* = < 0.05
Hayes et al. (2015) [61] *USA CS* *Study number in pinwheel* = *15*	*n* = 31, Mage = 64.5 (7.0), %*F* = 58, SB time = 565 Population: non-clinical	**Device** (Actigraph)	**Episodic Memory** (BVMT-Revised and the Faces subtest from the Wechsler Memory Scale-Third Edition, verbal memory Z score, CVLT logical memory recall) **Executive Function** (TMT, VF from the Delis Kaplan Executive Function System, DSB and mental arithmetic from WAIS and the WCST)	Age, gender, education, depression, hypertension, wear time	**SB and cognition** *Visuospatial memory test* pr = −0.41, *p* < 0.05 *face-name memory task* pr = −0.53, *p* < 0.05 *verbal memory*: nr *Executive Function:* nr
Hubbard et al. (2015) [79] *USA CS* *Study number in pinwheel* = *16*	*n* = 82, Mage = 49.0 (9.1), %F = 76, SB time = 582 Population: clinical	**Device** (Actigraph)	**Processing Speed** (SDMT)	Not reported	**SB and cognition** *overall:* *r* = −0.12, p = 0.29 *mild:* *r* = −0.14, p = 0.38 *moderate:* r = 0.06, p = 0.71
Iso-Markku et al. (2018) [78] *Finland CS* *Study number in pinwheel* = *17*	*n* = 726, Mage = 72.9 (1.0), %*F* = 52, SB time = 537 Population: non-clinical	**Device** (Hookie AM20)	**Global Cognitive Function** (Telephone assessment and interview for Cognitive Status (﻿orientation, serial subtraction, word recall, semantics, sentence repetition, linguistic skills, and attention)	Model 1: age, sex, accelerometer wear time, mean daily MET Model 2: age, sex, accelerometer wear time, BMI, living condition, years of education, mean daily MET	**Total cog score and SB *Between-family analyses**** β* (95% CI) *Model 1:* − 0.20 (− 0.41 to 0.01), *p* > 0.05 *Model 2: − *0.21 (0.42 to − 0.003), *p* < 0.05
Johnson et al. (2016) [65] *Australia CS* *Study number in pinwheel* = *18*	*n* = 188, Mage = 64.0 (7.3), %*F* = 54, SB time = 582 Population: non-clinical	**Device** (ActiGraph)	**Processing Speed** (TMT A) **Cognitive Flexibility & Working Memory** (TMT B) **Episodic Memory** (HVLT-R)	age, gender, level of education, waist to hip ratio, history of cigarette smoking, alcohol intake, HVLT total recall score (screening tool for MCI)	**SB and cognitive function** *TMT A: r* = 0.082, *p* = ns *TMT B: r* = 0.050, *p* = ns *HVLT: r* = 0.001 *p* = ns
Kojima et al. (2019) [40] Japan LO Study *number* in pinwheel = 43	*n* = 15, Mage = 78.0 (11.6), %*F* = 40 SB time = 1312 Population: clinical	**Device** (ActiGraph)	**Processing Speed** (SDMT) **Working Memory** (Symbol Trails, Design Memory) **Cognitive Flexibility** (Symbol Trails, Symbol Cancellation) **Executive Function** (Symbol Cancellation, Mazes)	Not stated	4 months Value of sedentary behavior significantly decreased over four months (*p* < 0.05) Less sedentary behavior was significantly correlated with better SDMT scores (*r* = −0.355, *p* = 0.005)
Koolhaas et al. (2019) [37] *Netherlands CS/LO* *Study number in pinwheel* = *19*	*n* = 1841, Mage = 62.6 (9.3), %*F* = 54, SB time = 528 Population: non-clinical	**Device** (Actigraph)	**Global Cognitive Function** (MMSE, g-factor test battery: MMSE, Stroop, letter-digit substitution task, VF15-word learning test, Purdue pegboard test)	Model 1: age, sex, cohort, awake time Model 3: age, sex, cohort, awake time, education, occupational status, marital status, smoking, BMI, PA and disability score	***Cross-sectional**** β [95% CI]* *SB and g-factor* *Model 1: − *0.03 [− 0.05, − 0.01], *p* = 0.005 *Model 3: − *0.01 [− 0.03, 0.01], *p* = 0.23 *SB and MMSE* *Model 1: − *0.01 [− 0.06, 0.04], *p* = 0.66 *Model 3: − *0.0004 [− 0.05, 0.05], *p* = 0.98 ***5.7-year follow-up*** *g-factor:* 0.18-point (SD: 0.51) decline *MMSE score:* 0.06-point decline (SD: 1.89)
Ku et al. (2017) [36] *Taiwan CS/LO* *Study number in pinwheel* = *20*	*n* = 274, Mage = 74.5 (6.1), %*F* = 54, SB time = categories Population: non-clinical	**Device** (Actigraph)	**Global Cognitive Function** (AD8)	Model 1: baseline cognitive status, sex, age, and wear time of accelerometer Model 3: baseline cognitive status, sex, age, years of formal education, marital status, income source, smoking, number of comorbidities, depressive symptoms, wear time of accelerometer, moderate-to-vigorous physical activity, and activities of daily living	***SB time & cognitive ability (baseline)*** *r* = 0.15, *p* = < 0.05 ***SB time & cognitive ability (2-year follow-up****)* *r* = 0.21, *p* = < 0 .001 ***Adjusted rate ratio [95% CI]*** *Model 1:* 1.13 [1.04, 1.22), *p* = 0.002 *Model 3:* 1.10 [1.00, 1.19], *p* = 0.047
Lee et al. (2013) [39] *Japan* LO *Study number in pinwheel* = *21*	*n* = 550, Mage = nr, %*F* = 48, SB time = nr Population: non-clinical	**Self-report** (Trained interviewers asked subjects about time spent in physical activity for the past 12 months)	**Global Cognitive Function** (MMSE) Incidence and Odds of Significant cognitive decline (− 3 points on MMSE)	Model 1: age, sex, educational level Model 3: age, sex, educational level, BMI, initial MMSE score, smoking status, self-rated health, Depression, sleep duration, whether participant was working, hypertension, myocardial infarction, hyperlipidemia, diabetes mellitus, stroke, rheumatoid arthritis and MVPA	**Longitudinal (8-years)** **OR [95% CI]** *Model 1*: 1.97, 95% [1.01, 3.86] *Model 3*: 3.03 [1.29, 7.14]
Leung et al. (2017) [76] *Canada CS* *Pattern* *Study number in pinwheel* = *22*	*n* = 114, Mage = 86.7 (7.5), %*F* = 85, SB = nr Population: non-clinical	**Device** (ActiGraph)	**Global Cognitive Function** (MoCA)	Not reported	***% waking time in SB and MoCA:*** *p* > 0.05
Lopes et al. (2015) [82] *Brazil CS* *Study number in pinwheel* = *23*	*n* = 2471, Mage = nr, %*F* = 60, SB time = categories Population: mixed	**Self-report** (The International physical activity questionnaire)	**Global Cognitive Function** (MMSE) ≤ 25 = low cognitive performance (LCP) > 25 = high cognitive performance	Not reported	**LCP prevalence [95% CI]** *1st tertile (*< *180 min/day):* 38.20 [32.10, 44.69] *2nd tertile (*> *180* < *308.61 min/day):* 28.42 [22.70, 34.93] *3rd tertile (*> *308.61 min/day):* 29.34 [24.48, 34.72] **LCP Prevalence ratio (PR) [95% CI]** *2nd tertile [*> *180* ≤ *308.61 min/day]* Crude PR, 0.74 [0.60, 0.91] Adjusted PR, 0.73 [0.59, 0.89], *p* < 0.05 *3rd tertile [*> *380.61 min/day]* Crude PR, 0.76 [0.62, 0.93] Adjusted PR, 0.75 (0.61–0.91), *p* < 0.05
Lu et al. (2018) [84] *Hong Kong* CS *Prevalence Pattern* *Study number in pinwheel* = *24*	*Healthy*: *n* = 271, Mage = 81.9 (3.5), %*F* = 38 *Low MoCA*: *n* = 252, Mage = 83.4 (4.0), %*F* = 48 *MCI: n* = 105, Mage = 83.6 (3.7), %F = 49 *AD*: *n* = 182, Mage = 80.8 (5.9), %*F* = 65 Population: mixed	**Device** (Actigraph)	﻿**Global Cognitive Function** (Hong Kong version of (MoCA)	Model 1: age, gender, wear time Model 2: age, gender, wear time, years of education, BMI, unusual gait speed, living status, disease burden	**Time in SB (min/day):** Controls = 546.7 Low MoCA = 534.1 MCI = 516.9 AD = 601.2
Maasakkers et al. (2020) [38] *Australia, USA, Japan, and Singapore* CS/LO *Study number in pinwheel* = *25*^a,b,c,d^	*PATH Cohort* *n* = 1552, Mage = 75.1 (1.5), %*F* = 49, SB time = 426 Population: non-clinical 25^a^	**Self-report** (asked two questions relating to SB on a usual day, which distinguished between weekdays and weekend days)	**Global Cognitive Function** (MMSE)	Unadjusted model: none Model 3: age, gender, education, income, alcohol consumption, smoking, BMI, marital status, living status, perceived health, morbidities, blood pressure, sleep quality, depression and PA	***Cross-sectional*** *Unadjusted* *B* = −0.003 [− 0.005, 0.001], *p* = 0.79 *Model 3* *B* = 0.001 [− 0.021, 0.022], p = 0.96
	*SALSA Cohort* *n* = 1663, Mage = 70.2 (6.8), %*F* = 58, SB time = 276 Population: non-clinical 25^b^	**Self-report** (administered three questions of SB related to sitting at work, at home, and while driving a car during a regular week)	**Global Cognitive Function** (3MS)	Unadjusted model: none Model 2: age, gender, ethnicity, education, income, alcohol consumption, smoking, BMI, marital status, living status, perceived health, morbidities, blood pressure, sleep quality, depression and PA	***Cross-sectional (B)*** *Unadjusted:* 0.33 [0.027, 0.632], *p* = 0.03 *Model 2*: − 0.043 [− 0.317, 0.230], *p* = 0.76 ***Longitudinal (8.1-year follow-up)*** *Unadjusted:* 0.008 [− 0.038, 0.053], *p* = 0.74 *Model 2*: − 0.011 [− 0.058, 0.037], *p* = 0.66
	*SGS Cohort* *n* = 2597, Mage = 73.4 (6.1), %*F* = 56, SB time = 444 Population: non-clinical 25^c^	**Device** (Active style Pro HJA-350IT)	**Global Cognitive Function** (MMSE)	Unadjusted model: none Model 2: age, gender, education, income, alcohol consumption, smoking, BMI, living status, perceived health, morbidities, depression and PA	***Cross-sectional (B)*** *Unadjusted: − *0.005 [− 0.015, 0.004], *p* = 0.25 *Model 2*: 0.006 [− 0.006, 0.018], *p* = 0.35 ***Longitudinal (2-year follow-up)*** *Unadjusted: − *0.003 [− 0.009, 0.004], *p* = 0.40 *Model 2*: − 0.001 [− 0.010, 0.007], *p* = 0.73
	*SLAS2 Cohort* *n* = 3087, Mage = 66.7 (7.8), %*F* = 63, SB time = 366 Population: non-clinical 25^d^	**Self-report** (asked two questions relating to SB on a usual day, which distinguished between weekdays and weekend days)	**Global Cognitive Function** (MMSE)	Model 1: unadjusted Model 2: age, gender, ethnicity, education, alcohol consumption, smoking, BMI, marital status, living status, perceived health, morbidities, blood pressure, sleep quality, depression and PA	***Cross-sectional (B)*** *Unadjusted:* 0.04 [− 0.004, 0.083], *p* = 0.08 *Model 2*: 0.118 [0.075, 0.160], *p* = < 0.001 ***Longitudinal (3.8-year follow-up)*** *Unadjusted: − *0.007 [− 0.021, 0.007], *p* = 0.32 *Model 2*: − 0.011 [− 0.027, 0.004], *p* = 0.16
Marinac et al. (2019) [64] *USA CS Pattern* *Study number in pinwheel* = *26*	*n* = 30, Mage = 62.2 (7.8), %*F* = 100, SB time = 498 Population: clinical	**Device** (activPAL)	**Cognitive Flexibility** (The Dimensional Change Card Sort Test) **Executive Function** (FLA) **Episodic Memory** (Picture Sequence Memory Test) **Working Memory** (List Sorting) **Processing Speed** (Pattern Comparison Test)	device wear time, education, employment status, MVPA, chemotherapy status	**Total sitting time: (b, p)** *Executive Function:* 0.21, 0.88 *Cognitive Flexibility:* − 2.75, 0.06 *Episodic memory:* 2.69, 0.34 *Working memory:* − 1.01, 0.63 *Processing speed: − *2.47, 0.32
Olanrewaju et al. (2020) [80] *Ireland CS* *Study number in pinwheel* = *47*	*n* = 8163, Mage = 63.5 (9.2), %F = 52, SB time = 295 Population: non-clinical	**Self-report** (International Physical Activity Questionnaire (IPAQ))	**Processing Speed** (AF) **Global Cognitive Function** (MMSE) **Episodic Memory** (Immediate and delayed recall)	Age, sex, social class	**Episodic memory:** *β* **(95% CI)** 0.01 (− 0.004, 0.02), *p* > 0.05 **Processing Speed:** *β* **(95% CI)** 0.003 (− 0.01, 0.01), *p* > 0.05 **Global Cognitive Function:** *β* **(95% CI)** 0.01 (− 0.01, 0.02), *p* > 0.05
Rosenberg, et al. (2016) [51] *USA CS* *Study number in pinwheel* = *27*	*n* = 307, Mage = 83.6 (6.4), %*F* = 72, SB time = 516 Population: non-clinical	**Device** (ActiGraph)	**Working Memory & Cognitive Flexibility** (TMT B) **Processing Speed** (TMT A)	age, gender, marital status, educational status, MVPA, wear time	**Objective Sedentary Time (*β (SE))*** *TMT A:* − 0.02 (0.02) *p* = 0.33 *TMT B:* − 0.03 (0.02) *p* = 0.18
	*n* = 280, SB time = 660 Population: non-clinical	**Self-report** (Sedentary Behaviour Questionnaire)	**Working Memory & Cognitive Flexibility** (TMT B) **Processing Speed** (TMT A)	age, gender, marital status, educational status, MVPA	**Self-reported SB (*β(SE))*** *TMT A:* − 0.01 (0.00) *p* = < 0.01 *TMT B*: 0.01 (0.01) *p* = 0.08
Siddarth et al., (2018) [81] *USA CS* *Study number in pinwheel* = *28*	*n* = 35, Mage = 60.4 (8.1), %*F* = 71, SB time = 432 Population: non-clinical	**Self-report** [International Physical Activity Questionnaire modified for older adults (average number of hours spent sitting)]	**Episodic Memory** (Verbal paired association, Selective reminding scores) **Processing Speed** (Digit symbol scores)	age	*Verbal paired association: r* = 0.12 *p* = 0.5 *Selective reminding scores: r* = 0.28 *p* = 0.11 *Digit symbol scores: r* = −0.18 *p* = 0.34
Snethen et al. (2014) [44] *USA CS 29* *Study number in pinwheel* = *29*	*n* = 30, Mage = 50.6 (nr), %*F* = 10, SB time = 406 Population: clinical	**Device** (Actigraph)	**Cognitive Flexibility** (WCST)	Diagnosis, sex, age, BMI	*r* = 0.04, *p* > 0.05
Stubbs et al. (2017) [56] *Taiwan CS* *Study number in pinwheel* = *30*	*Schizophrenia* *n* = 199, Mage = 44.0 (9.9), %*F* = 39, SB time = 581 *Controls* *n* = 60, Mage = 41.9 (9.6)*,* %*F* = 43, SB time = 336 Population: mixed	**Device** (ActiGraph)	**Processing Speed** (COG) **Motor Skills and Construction** (Reaction Test, GPT)	Model 1: age, sex, education, weight status, smoking, alcohol consumption, medications, PANSS, MetS Model 2: age, sex, education, weight status, smoking, alcohol consumption, medications, PANSS, MetS, Physical activity energy expenditure	**Total SB and cognitive function outcomes** *Schizophrenia group* *COG: p* = *0.403* *GPT****: p* = *0.020*** *Reaction Time reaction (msec): p* = *0.984* *Reaction Time motor (msec): p* = *0.070* *control group* *COG: p* = *0.295* *GPT: ****p* = *0.016*** *Reaction Time reaction (msec): p* = *0.016* *Reaction Time motor (msec): p* = *0.378* **Comparing means b/w low and high SB in Patients with Schizophrenia** *Reaction Time reaction (msec) p* = 0.803 669.5 (SD = 532.2) low sed 652.3 (SD = 410.0) high sed *Reaction Time motor (msec****), p* = 0.037** 355.2 (SD = 170.8) low sed 421.3 (SD = 252.7) high sed *COG*, *p* = 0.442 176.9 (SD = 95.1) low sed 165.8 (SD = 107.1) high sed *GPT: ****p* = 0.034** 131.6 (SD = 44.1) low sed 145.4 (SD = 46.7) high sed
Suzuki et al. (2020) [57] *Japan CS Prevalence* *Study number in pinwheel* = *48*	*Males* *n* = 68, Mage = 88.0 (1.0), %*F* = 0, SB time = 855 *Females* *n* = 68, Mage = 88.0 (0.9), %*F* = 100, SB time = 798 Population: mixed	**Device** (Actigraph)	**Global Cognitive Function** (ACE) Score of ≤ 88 indicating cognitive impairment	Single Factor Model: device wear time, age, education and the Center for Epidemiologic Studies Depression Scale Partition Model: All time units spent performing any of the activity categories and covariates were entered into the same model, and the independent effects of each behavioral variable were examined	**Males** *Time in SB and cognitive function* *β* = −0.069, *p* = 0.332 **Females** *Time in SB and cognitive function* *β* = −0.026, *p* = 0.758
Vancampfort et al. (2018) [52] *China, Ghana, India, Mexico, Russia, and South Africa CS* *Study number in pinwheel* = *31*	*Whole sample: n* = 32,715, Mage = 62.1 (15.6), %*F* = 50 *MCI* *n* = 4082, Mage = 64.4 (17.0), %*F* = 55, SB time = 262 Population: mixed	**Self-report** (Global physical activity questionnaire)	**Global Cognitive Function** (Based on the recommendations of the National Institute on Aging-Alzheimer’s Association)	sex, age, years of education, wealth, depression, obesity, number of chronic conditions, low PA, country	***OR [95% CI]*** ≥ *8 h vs* < *8/day SB and cognitive function* 1.56 [1.27, 0.91], *p* < 0.001 *1 h increase in SB* 1.08 [1.05–1.11], *p* < 0.001
Vance et al. (2016) [49] *USA CS* *Study number in pinwheel* = *32*	*n* = 122, Mage = 70.5 (7.2), %*F* = 57, SB time = 803 Population: non-clinical	**Self-report** (Single item from the Physical activity questionnaire)	**Processing Speed** (AF, TMT A) **Episodic Memory** (CVLT-II) **Working Memory & Cognitive Flexibility** (TMT B)	Not reported	**Correlations with SB** AF: *r* = −0.09, *p* = ns *CVLT-II*: *r* = 0.00, *p* = ns *TMT A*: *r* = 0.10, *p* = ns *TMT B*: *r* = −0.03, *p* = ns
Vasquez et al. (2017) [47] *USA CS* *Study number in pinwheel* = *33*	*n* = 7478, age range: 45–75, %*F* = 62, SB time = 738 Population: non-clinical	**Device** (Actical model 198–0200-06) **Self-report** (not reported)	**Global Cognitive Function** (cognitive function overall score) **Episodic Memory** (B-SEVLT) **Processing Speed** (Word fluency, DSST)	Sex	**Device measured SB (each 10 min/day increase) *β* (SE):** *Cognitive Function overall score:* − 0.044 (0.006), *p* < 0.0001 *Word Fluency*: − 0.004 (0.002), *p* = 0.0356 *DSST*: − 0.198 (0.022), *p* < 0.0001 *SEVLT Sum3 trials: − *0.033 (0.005), *p* < 0.0001 *SEVLT Free recall:* − 0.033 (0.005), *p* < 0.0001 **Self-reported sedentary time (each 10 min/day increase):** *Cognitive Function overall score:* 0.019 (0.003), *p* < 0.0001 *Word Fluency:* 0.006 (0.001), *p* < 0.0001 *DSST:* 0.115 (0.014), *p* < 0.0001 *SEVLT Sum3 trials:* − 0.033 (0.005), *p* = 0.1154 *SEVLT Free recall:* 0.005 (0.003), *p* = 0.0583
Wanigatunga et al. (2018) [53] *USA CS Pattern* *Study number in pinwheel* = *34*	*n* = 1275, Mage = 79 (5.0), %*F* = 67, SB time (min–max) = 24–512 Population: non-clinical	**Device** (ActiGraph)	**Processing Speed** (DSC) **Episodic Memory** (HVLT-Revised) **Working Memory** (*n*-back) **Cognitive Flexibility** (Task switching paradigm) **Executive Function** (FLA) **Global Cognitive Function** (global composite: DSC, HVLT, n-back, task switching paradigm)	age, sex, race/ethnicity, education, income, marital status, BMI, smoking status, sleep quality, perceived stress, living with two or more morbid conditions	**Associations b/w low and high total SB, *β* (SE)** *one-back high:* − 0.012 (0.013) t*wo back high*: 0.001 (0.016) *DSC high*: − 2.03 (0.854), *p* < 0.05 *task-switching (no) high:* 86.22 (74.541) *task-switching (yes) high:* 117.953 (94.122) *FLA congruent high:* 24.541 (15.994) *FLA incongruent high:* 18.602 (23.158) *HVLT immediate high:* 0.385 (0.363) *HVLT delayed high:* 0.252 (0.197) *global cognitive function:* 0.085 (0.047)
Watts et al. (2018) [54] *USA CS Prevalence Pattern* *Study number in pinwheel* = *35*	*Mild AD* *n* = 47, Mage = 73.1 (8.0), %*F* = 34, SB time = 584 *Controls* *n* = 53, Mage = 73.2 (6.5), %*F* = 69, SB time = 556.8 Population: mixed	**Device** (activPAL)	**Global Cognitive Function** (MMSE) **Processing Speed** (WAIS-DSST, block design, digits forward, AF, vegetable fluency, TMT A) **Executive Function** (Stroop) **Cognitive Flexibility** (Letter number sequencing, TMT B) **Working Memory** (Letter number sequencing, DSB, TMT B) **Episodic Memory** (logical memory immediate, logical memory delayed) Mild AD diagnosis: = 0.5 (very mild) or 1 (mild) Controls: = 0 (no dementia)	None	**(whole sample, *n* = 83):** *MMSE: r* = −0.082 *WAIS: r* = −0.053 *Block Design: r* = 0.044 *Stroop Interference: r* = −0.129 *Letter Number Sequencing: r* = 0.139 *Logical Memory Immediate: r* = −0.285, *p* = 0.015 *Logical Memory Delayed: r* = −0.267, *p* = 0.022 *Digits Forward: r* = −0.011 *Digits Backward: r* = 0.000 *Animal fluency: r* = −0.156 *Vegetable fluency: r* = −0.165 *TMT A: r* = 0.093 *TMT B: r* = 0.148
Wei et al. (2021) [43] *USA CS* *Isotemporal* *Study number in pinwheel* = *52*	*Sleep* ≤ *7 h per night* *n* = 1843, Mage = nr %*F* = ~ 50, SB time = nr *Sleep* > *7 h per night* *n* = 1243, Mage = nr %*F* = ~ 43, SB time = nr Population: non-clinical	**Self-report** (The Global Physical Activity Questionnaire) Isotemporal: Replacing sleep, sedentary activity, walking/bicycling, MVPA with each other	**Episodic Memory** (CERAD Word Learning subtest) **Processing Speed** (DSST, AF)	age, sex, race/ethnicity, education level, smoking, and body mass index	***Sleep* ≤ *7 h per night***; ***β (95% CI)*** *DSST:* 0.002 (− 0.01, 0.01) *CERAD:* 0.01 (0.003, 0.02), *p* < 0.05 *AF:* 0.01 (0.003, 0.02), *p* < 0.05 ***Sleep* > *7 h per night; β (95% CI)*** *DDST:* 0.003 (− 0.01, 0.01) *CERAD*: 0.01 (− 0.003, 0.02) *AF*: 0.003 (− 0.01, 0.02) Among participants with sleep duration ≤ 7 h/night, replacing 30 min/day of sedentary activity with 30 min/day of MVPA or 30 min/day was associated with better cognition. Among participants with sleep duration > 7 h/night, replacing 30 min/day of sleep with 30 min/day of sedentary activity, walking/bicycling, or MVPA was associated with better cognition
Wu et al. (2020) [63] *China CS* *Study number in pinwheel* = *53*	*n* = 308, Mage = 68.66 (5.37), %*F* = 57, SB time = 591 Population: non-clinical	**Device** (Actigraph)	**Global Cognitive Function** (MoCA)	Model 1: uncorrected Model 2: age, BMI, highest education, monthly average income. SED sedentary behavior, LPA light physical activity, MVPA moderate-vigorous physical activity, TPA total physical activity	***SB and Global Cognitive Function*** *Model 1:* ***β*** = −0.020 SE = 0.001, *p* = 0.061 *Male subgroup* ***β*** = −0.003 SE = 0.001 *p* = 0.029
Zhu, W. et al. (2015) [67] *USA CS* *Study number in pinwheel* = *38*	*n* = 7098, Mage = 70.1 (8.5), %*F* = 54.2, SB time = nr Population: clinical	Device (Actical)	**Episodic Memory** (word list learning, recall) **Processing Speed** (semantic fluency, letter fluency) **Global Cognitive Function** (items from MOCA)	Model 1 was unadjusted Model 3 was adjusted for age, sex, race, region of residence, education, ST%, BMI, hypertension, smoking, and diabetes mellitus	*Not reported*
Zlatar (2019) [66] USA*CS* *Study number in pinwheel* = *37*	*n* = 52, Mage = 72.3 (5.0), %*F* = 57.7, SB time = 548 Population: non-clinical	**Device** (ActiGraph)	**Processing Speed** (Letter fluency) **Executive Function** (colour word inhibition **Working Memory** (TMT B) **Cognitive Flexibility** (TMT B, WCST) **Episodic Memory** (Face naming score, CVLT, WMS-R)	Unadjusted	*CWI switch: r* = 0.118, *p* > 0.05 *CWI: r* = −0.011, *p* > 0.05 *letter fluency: r* = −0.129, *p* > 0.05 *TMT B: r* = −0.087, *p* > 0.05 *WCST: r* = 0.036, *p* > 0.05 *face naming score: r* = −0.232, *p* > 0.05 *CVLT-II List A: r* = 0.195, *p* > 0.05 *CVLT-II short delay: r* = 0.184, *p* > 0.05 *CVLT-II long delay: r* = 0.187, *p* > 0.05 *WMS-R LMI: r* = 0.248, *p* > 0.05 *WMS-R LMII: r* = 0.254, *p* > 0.05

*ACE* Addenbrooke’s Cognitive Examination, *AF* Animal Fluency, *AOS* Automated Operation Span, *B-SEVLT* Brief Spanish English Verbal learning Test, *BVMT* Brief Visuospatial Memory Test, *CVLT* California Verbal Learning Test, *AD8* Chinese version of the Ascertain Dementia 8-item questionnaire, *COG* Cognitrone Test, *CS* Cross Sectional, *DSB* Digit Span Backwards, *DSC* Digit Symbol Coding, *DSST* Digit Symbol Substitution Task, *FLA* Flanker or Eriksen Flanker Test, *GPT* Grooved Pegboard Test, *HVLT* Hopkins Verbal Learning Test, *LO* Longitudinal, *MMSE* Mini-Mental State Examination, *MoCA* Montreal Cognitive Assessment, *nr* Not reported, *PASAT* Paced Auditory Serial Addition Test, *RT* Reaction Time, *SB* Sedentary Behaviour, *SDMT* Symbol Digit Modalities Test, TMT *A* Trail Making Test A, *TMT B* Trail Making Test B, *VF* Verbal Fluency, *WAIS* Wecshler Adult Intelligence Scale, *WMS-R* Wechsler Memory Scale-revised, *WCST* Wisconsin Card Sorting Test, *3MS* Modified Mini Mental State Examination

**Table 2:** Summary and characteristics of cross-sectional studies reporting the associations for pattern of sedentary behaviour accumulation with cognitive function

Authors (year) *Country* *Study Design* *Pinwheel number*	Participants Mean age (Mage) % F (female) SB time (min)	Device or self-report (measure of sedentary behaviour)	Domain (outcome measure)	Covariates adjusted for	Results	Conclusion
Bollaert et al. (2019) [68] *USA CS Prevalence Pattern* *Study # in pinwheel* = *39*	*Healthy group* *n* = 40, Mage = 66.5 (6.7), %*F* = 62.5, SB time = 534 *Multiple Sclerosis group* *n* = 40, Mage = 65.3 (4.3), %*F* = 62.5, SB time = 540 Population: mixed	**Device** (Actigraph)	**Processing Speed** (SDMT, PASAT) **Episodic Memory** (BVMT, CVLT-II)	Not stated	***# of SB bouts*** ***Between groups*** *p* > 0.05 ***Healthy Controls (r)*** SDMT: − 0.12 CVLT-II: − 0.17 BVMT-R: − 0.04 PASAT: − 0.08 ***MS group (r)*** SDMT: 0.09 CVLT-II: 0.24 BVMT-R: 0.08 PASAT: 0.20 ***Duration of SB bouts:*** ***Between groups*** *p* > 0.05 ***Healthy Controls (r)*** SDMT: − 0.11 CVLT-II: − 0.10 BVMT-R: 0.10 PASAT: 0.01 ***MS group (r)*** SDMT: − 0.22 CVLT-II: 0.01 BVMT-R: − 0.08 PASAT: − 0.18 ***# of long SB bouts (*> *30 min)*** ***Between groups*** *p* > 0.05 ***Healthy Controls (r)*** SDMT: − 0.08 CVLT-II: − 0.27 BVMT-R: 0.02 PASAT: − 0.09 ***MS group (r)*** SDMT: − 0.17 CVLT-II: 0.22 BVMT-R: − 0.02 PASAT: 0.03 ***Duration of long SB bouts*** ***Between groups*** *p* < 0.015 ***Healthy Controls (r)*** SDMT: − 0.21 CVLT-II: − 0.02 BVMT-R: 0.11 PASAT: − 0.01 ***MS group (r)*** SDMT: − 0.05 CVLT-II: 0.04 BVMT-R: − 0.02 PASAT: 0.08	**Pattern of SB was not associated with cognitive function** **Duration of long sedentary bouts were longer in the MS group compared to the controls**
Cukic et al. (2018) [35] *Scotland* *CS/LO* *Pattern* *Study # in pinwheel* = *3*^a,b,c^	LBC1936 cohort *n* = 271, Mage = 79.0 (0.4) % *F* = 48.3, SB time = 626.8 Population: non-clinical 3^a^	**Device** (activPal3)	**Global Cognitive Function** (general cognitive ability factor (g) computed from 6 tests taken from the ﻿WAIS (﻿Matrix Reasoning, Block Design, Letter-Number Sequencing, Symbol Search, DSB, and Digit Symbol), Moray Houst Test No. 12 (MHT), Alice Heim 4 test (AH4)) **Processing Speed** Four-choice (RT) **Motor Skills and Construction** (Simple RT)	Model 1: age and sex Model 3: age, sex, education, long standing illness	**Interruptions: *β*, [95% CI]** *Model 1* *g-factor:* 0.02 [− 0.10, 0.14], *p* = 0.80 *Simple RT:* 0.00 [− 0.12, 0.12], *p* = 0.99 *Choice RT: − *0.03 [− 0.15, 0.09], *p* = 0.60 *MHT Age 11:* 0.07 [− 0.05, 0.19], *p* = 0.24 *MHT change age 11–79:* 0.03 [− 0.11, 0.14], *p* = 0.99 *Model 3* *g-factor:* − 0.04 [− 0.18, 0.10], *p* = 0.61 *Simple RT:* 0.01 [− 0.11, 0.13], *p* = 0.89 *Choice RT:* 0.03 [− 0.15, 0.09], *p* = 0.60 *MHT change age 11–79:* 0.01 [− 0.01, 0.03], *p* = 0.99	**Interruptions in SB were not associated with cognitive function**
	Twenty-07 1950’s cohort *n* = 310, Mage = 64.6 (0.9) %*F* = 53.2, SB time % = 60.8 Population: non-clinical 3^b^			Model 1: age and sex Model 4: age, sex, education, long standing illness, employment status	**Interruptions: *β*, [95% CI]** *Model 1* *AH4 wave 5:* 0.05 [− 0.07, 0.17], *p* = 0.37 *Simple RT wave 5: − *0.06 [− 0.18, 0.06], *p* = 0.27 *Choice RT wave 5:* − 0.04 [− 0.16, 0.08], *p* = 0.49 *Model 4* *AH4 wave 5:* 0.11 [− 0.03, 0.25], *p* = 0.11 *Simple RT wave 5: − *0.06 [− 0.06, 0.18], *p* = 0.29 *Choice RT wave 5: − *0.05 [− 0.17, 0.07], *p* = 0.43	**Interruptions in SB were not associated with cognitive function**
	Twenty-07 1930’s cohort *n* = 119, Mage = 83.4 (0.6) % *F* = 54.6, SB time % = 68.2 Population: non-clinical 3^c^			Model 1: age and sex Model 3: age, sex, education, long standing illness	**Cog ability & SB interruptions: *β*, [95% CI]** *Model 1* *AH4 wave 1:* 0.08 [− 0.10, 0.26], *p* = 0.41 *AH4 wave 5:* 0.05 [− 0.15, 0.25], *p* = 0.60 *Simple RT: − *0.07 [− 0.27, 0.13], *p* = 0.47 *Choice RT:* 0.09 [− 0.09, 0.27], *p* = 0.32 *Model 3* *AH4 wave 1:* 0.13 [− 0.09, 0.35], *p* = 0.24 *AH4 wave 5:* 0.10 [− 0.12, 0.32], *p* = 0.41 *Simple RT: − *0.09 [− 0.29, 0.11], *p* = 0.39 *Choice RT:* 0.04 [− 0.21, − 0.29], *p* = 0.77	**Interruptions in SB were not associated with cognitive function**
English et al. (2016) [74] *Australia* *CS Pattern* *Study # in pinwheel* = *7*	*n* = 50, Mage = 67.2 (11.6) % *F* = 34.0, SB = nr Population: clinical	**Device** (activPAL)	**Global Cognitive Function** (MoCA)	Waking hours	**MoCA with prolonged sitting time (≥ 30)** *r* = −0.006, *p* = 0.970	**Prolonged sitting was not associated with cognitive function**
Ezeugwu et al. (2017) [75] *Canada CS Pattern* *Study # in pinwheel* = *8*	*n* = 30, Mage = 63.8 (12.3) % *F* = 43.3, SB time = 673.9 Population: clinical	**Device** (activPAL)	**Global Cognitive Function** (MoCA)	Not reported	**Sedentary interruptions and MoCA** *r* = 0.07, *p* > 0.05	**Interruptions in SB time were not associated with cognitive function**
Falck et al. (2017) [55] *Canada CS Prevalence Pattern* *Study # in pinwheel* = *10*	*Probable MCI* *n* = 81, Mage = 72.5 (7.6) % *F* = 59.8, SB time = 594.8 *Without MCI* *n* = 69, Mage = 69.4 (6.4) % *F* = 77.9, SB time = 541.6 Population: mixed	**Device** (MotionWatch8)	**Global Cognitive Function** (MoCA, ADAS-Cog Plus) Probable MCI = MoCA < 26	Age, sex, education	**Mean (SD) b/n those with MCI & without** *Average 30* + *min bouts/day SB* with probable MCI = 4.07 (1.85)without MCI = 3.30 (1.73) *p* = 0.046 **SB and ADAS-Cog Plus (*β***) *Average 30* + *min bouts/day*: 0.061, *p* = 0.016 **SB and ADAS-Cog Plus Based on MCI Status (*β***) *non-MCI* Average 30 + min bouts/day: 0.075, *p* = 0.064 *MCI* Average 30 + min bouts/day: 0.033, *p* = 0.282	**Participants with probable MCI had more 30 + min bouts/day of SB compared to those without MCI** **Significant association between greater 30 + min bouts/day of SB and poorer cognitive performance** **Marginal relationship between greater 30 + min bouts/day of SB and poorer cognitive function for participants without MCI** **No relationship for more 30 + min bouts of SB and cognitive performance for those with probable MCI**
Hartman et al. (2018) [83] *Netherlands CS Prevalence Pattern* *Study # in pinwheel* = *N/A*	*Dementia* *n* = 45, Mage = 79.6 (5.9) % *F* = 51, SB time = 510 *Controls* *n* = 49, Mage = 80.0 (7.7) % *F* = 48.9, SB time = 486 Population: mixed	**Device** (Philips Actiwatch 2)	**Global Cognitive Function** (MMSE)	Not reported	**# of interruptions in SB (SD)** *Dementia*: 28.2 (26.2–32.5) *Control:* 27.2 (24.5–31.0) *p* = 0.195 **# of 30 min prolonged bouts (SD)** *Dementia*: 2.0 (0.9–3.3) *Control:* 2.3 (1.0–4.1) *p* = 0.227 **Duration of avg SB bout (SD)** *Dementia*: 16.6 (15.3–18.4) *Control:* 18.3 (16.4–21.1) *p* = 0.008	**No significant difference between groups for number of interruptions or number of 30-min prolonged bouts of SB** **The dementia patients had significantly longer durations of SB bouts compared to the controls**
Leung et al. (2017) [76] *Canada CS Pattern* *Study # in pinwheel* = *22*	*n* = 114, Mage = 86.7 (7.5) % *F* = 85.1, SB = 835 Population: non-clinical	**Device** (ActiGraph)	**Global Cognitive Function** (MoCA)	Not reported	***# of sedentary bouts:*** *p* > 0.05 ***Duration of sedentary bouts:*** *p* > 0.05	**Number and duration of SB bouts were not associated with cognitive function**
Lu et al. (2018) [84] *Hong Kong* CS *Prevalence Pattern* *Study # in pinwheel* = *24*	*Healthy*: *n* = 271, Mage = 81.9 (3.5), % *F* = 38.2 *Low MoCA*: *n* = 252, Mage = 83.4 (4.0), % *F* = 47.6 *MCI: n* = 105, Mage = 83.6 (3.7), % *F* = 48.6 *AD*: *n* = 182, Mage = 80.8 (5.9), % *F* = 65.4 Population: mixed	**Device** (Actigraph)	**Global Cognitive Function** (Hong Kong version of MoCA)	Model 1: age, gender, wear time Model 2: age, gender, wear time, years of education, BMI, unusual gait speed, living status, disease burden	**Average SB bout length compared to the AD group:** *Controls:* 6.6 (0.2), *p* < 0.05 *Low MoCA:* 6.5 (0.2), *p* < 0.05 *MCI:* 6.3 (0.3), *p* < 0.05 *AD:* 7.9 (0.2) **# of SB bouts > 30 min compared to the AD group = ***Controls:* 3.3 (0.1), *p* < 0.05 *Low MoCA*: 3.3 (0.1), *p* < 0.05 *MCI:* 3.5 (0.2), *p* < 0.05 *AD:* 4.1 (0.1)	**AD patients had longer SB bouts compared to the other 3 groups** **AD patients had more SB bouts > 30 min compared to the other 3 groups**
Marinac et al. (2019) [71] *USA CS Pattern* *Study # in pinwheel* = *26*	*n* = 30, Mage = 62.2 (7.8) % *F* = 100, SB time = 498 Population: clinical	**Device** (activPAL)	﻿**Cognitive Flexibility** (The Dimensional Change Card Sort Test) **Executive Function** (FLA) **Episodic Memory** (Picture Sequence Memory Test) **Working Memory** (List Sorting) **Processing Speed** (Pattern Comparison Test)	Device wear time, education, employment status, MVPA, chemotherapy status	**Time in sitting bouts > 20 min: (b, p)** *Executive Function:* − 0.73, 0.54 *Cognitive Flexibility:* − 2.82, 0.02 *Episodic memory*: 3.29, 0.17 *Working memory:* 1.36, 0.44 *Processing speed:* − 1.21, 0.57 **Sit-to-stand transitions: (b, p)** *Executive Function:* 0.14, 0.27 *Cognitive Flexibility****:*** 0.16, 0.2 *Episodic memory:* − 0.06, 0.82 *Working memory:* − 0.36, 0.051 *Processing speed:* 0.07, 0.77	**More time spent in prolonged sitting bouts was associated with worse cognitive flexibility scores** **More sit-to-stand transitions was not associated with cognitive function**
Wanigatunga et al. (2018) [53] *USA CS Pattern* *Study # in pinwheel* = *34*	*n* = 1275, Mage = 79 (5.0) % *F* = 67, SB time (min–max) = 24–512 Population: non-clinical	**Device** (ActiGraph)	**Processing Speed** (Digit Symbol Coding (DSC)) **Episodic Memory** (HVLT) **Working Memory** (*n*-back) **Cognitive Flexibility** (Task switching paradigm) **Executive Function** FLA **Global Cognitive Function** (DSC, HVLT, n-back, task switching paradigm)	Age, sex, race/ethnicity, education, income, marital status, BMI, smoking status, sleep quality, perceived stress, living with two or more morbid conditions	**Association’s b/w low and high 30 + min bouts of SB [b, (SE)]** *One-back high:* − 0.014 (0.013) *Two back high:* − 0.003 (0.016) *DSC high:* − 0.519 (0.879) *Task-switching (no) high:* 50.636 (76.635) *Task-switching (yes) high:* 27.604 (96.787) *Flanker congruent high:* 12.798 (16.416) *Flanker incon high:* 3.798 (23.759) *HVLT immediate high:* 0.284 (0.372) *HVLT delayed high:* 0.199 (0.202) *Global composite high:* − 0.012 (0.048)	**No significant associations for more 30 + min bouts of SB and cognitive function**
Watts et al. (2018) [54] *USA CS Prevalence Pattern* *Study # in pinwheel* = *35*	*Mild AD* *n* = 47, Mage = 73.1 (8.0) % *F* = 34. SB time = 584 *Controls* *n* = 53, Mage = 73.2 (6.5) % *F* = 69, SB time = 556.8 Population: mixed	**Device** (activPAL)	**Global Cognitive Function** (MMSE) Mild AD diagnosis: = 0.5 (very mild) or 1 (mild) Controls: = 0 (no dementia)	None	**# interruptions, (SD)** *Mild AD activPAL:* 42.28 (13.43) *Controls activPAL:* 47.52 (12.01) *p* = 0.06 **30 + min bouts** *Mild AD activPAL:* 5.49 (1.35) *Controls activPAL:* 4.91 (1.57) *p* = 0.07	**Number of SB interruptions or 30 + min bouts of SB did not differ between groups**

*ACE* Addenbrooke’s Cognitive Examination, *AD* Alzheimer’s Disease, *AF* Animal Fluency, *AOS* Automated Operation Span, *BVMT* Brief Visuospatial Memory Test, *CVLT* California Verbal Learning Test, *COG* Cognitrone Test, *DSB* Digit Span Backwards, *DSST* Digit Symbol Substitution Task, *FLA* Flanker or Eriksen Flanker Test, *GPT* Grooved Pegboard Test, *HVLT* Hopkins Verbal Learning Test, *Mage* Mean Age, *MMSE* Mini-Mental State Examination, *MoCA* Montreal Cognitive Assessment, *PASAT* Paced Auditory Serial Addition Test, *RT* Reaction Time, *SB* Sedentary Behaviour, *SDMT* Symbol Digit Modalities Test, *TMT A* Trail Making Test A, *TMT B* Trail Making Test B, *VF* Verbal Fluency, *WAIS* Wecshler Adult Intelligence Scale, *WCST* Wisconsin Card Sorting Test

**Table 3:** Summary and characteristics of cross-sectional studies reporting prevalence of sedentary behaviour for clinical and non-clinical populations

Authors (year) *Country* *Study Design* *Pinwheel number*	Participants Mean age (Mage) % F (female) SB time (min)	Device or self-report (measure of sedentary behaviour)	Domain (outcome measure)	Covariates adjusted for	Results	Conclusion
Amagasta et al. (2020) [77] *Japan CS Prevalence* *Study number in pinwheel* = *1*	*Cognitive decline* *n* = 48, Mage = 77.6 (5.4) % *F* = 52, SB time = 476.2 *Non-cognitive decline* *n* = 463, Mage = 73.0 (5.4), % *F* = 53, SB time = 442.4 Population: mixed	**Device** (Active style Pro HJA-750C)	**Global Cognitive Function** (MMSE) ≤ 23 = Cognitive Function Decline (CFD)	Model 1: unadjusted Model 4: Gender, age, education, BMI, living arrangements, working status, smoking, alcohol use, past history of stroke, medication for hypertension, dyslipidemia, diabetes	**SB (min) mean (SD)** **Cognitive Decline** 476.2 (153.9) **Non-Cognitive Decline** 442.4 (126.8) ***p* = 0.086**	**No significant difference in total SB time between the two groups**
Bollaert & Motl (2019) [68] *USA CS Prevalence Pattern* *Study number in pinwheel* = *39*	*Healthy group* *n* = 40, Mage = 66.5 (6.7) % *F* = 62.5, SB time = 534 *Multiple Sclerosis group* *n* = 40, Mage = 65.3 (4.3) % *F* = 62.5, SB time = 540 Population: mixed	**Device** (Actigraph)	**Processing Speed** (SDMT, Paced Auditory Serial Addition Test) **Episodic Memory** (Brief Visuospatial Memory Test-revised, CVLT-II)	Not stated	***Total SB*** ***Between groups*** *p* < 0.05 ***# of SB bouts*** ***Between groups*** *p* > 0.05 ***Duration of SB bouts:*** ***Between groups*** *p* > 0.05 ***# of long SB bouts (*> *30 min)*** ***Between groups*** *p* > 0.05 ***Duration of long SB bouts*** ***Between groups*** *p* < 0.015	**Total SB time was significantly greater in the impaired group** **Duration of SB bouts were significantly greater for impaired group**
Da Ronch et al. (2015) [46] *Italy Switzerland Germany CS Prevalence* *Study number in pinwheel* = *N/A*	*Whole sample* *n* = 1383 Mage = 73.1 (5.7), %*F* = 47.6 *MCI*: *n* = 251 *No cognitive impairment: n* = 1132 Population: mixed	**Self-report** (Self-report daily hours sitting)	**Global Cognitive Function** (MMSE) 18–26 = MCI 27–30 = No cognitive impairment	Gender, age, education, employment status, financial situation, living status, study centre	***mean hours/day (SD)*** MCI: 3.98 (SD = 1.42) No MCI: 3.62 (SD = 1.4) *p* < 0.001	**Total SB was significantly higher in the impaired group**
Falck et al. (2017) [55] *Canada CS Prevalence Pattern* *Study number in pinwheel* = *10*	*Probable MCI* *n* = 81, Mage = 72.5 (7.6) % *F* = 59.8, SB time = 594.8 *Without MCI* *n* = 69, Mage = 69.4 (6.4) % *F* = 77.9, SB time = 541.6 Population: mixed	**Device** (MotionWatch8)	**Global Cognitive Function** (MoCA, ADAS-Cog Plus) Probable MCI = MoCA < 26	Age, sex, education	**Mean (SD) b/n those with MCI & without** *% Sedentary time* with probable MCI = 61.65 (11.35) without MCI = 57.24 (12.38) *p* = 0.161 *Average 30* + *min bouts/day SB* with probable MCI = 4.07 (1.85) without MCI = 3.30 (1.73) *p* = 0.046	**Total SB time did not significantly differ between groups** **Impaired group had significantly more 30 + min bouts of SB/day compared to the non-impaired group**
Hartman et al. (2018) [83] *Netherlands CS Prevalence Pattern* *Study number in pinwheel* = *N/A*	*Dementia* *n* = 45, Mage = 79.6 (5.9) % *F* = 51, SB time = 510 *Controls* *n* = 49, Mage = 80.0 (7.7) % *F* = 48.9, SB time = 486 Population: mixed	**Device** (Philips Actiwatch 2)	**Global Cognitive Function** (MMSE)	Not reported	**SB minutes (SD)** *Dementia*: 510 (432–600) *Control: 486* (432–552) *p* = 0.0216 **# of interruptions in SB (SD)** *Dementia*: 28.2 (26.2–32.5) *Control:* 27.2 (24.5–31.0) *p* = 0.195 **# of 30 min prolonged bouts (SD)** *Dementia*: 2.0 (0.9–3.3) *Control:* 2.3 (1.0–4.1) *p* = 0.227 **Duration of avg SB bout (SD)** *Dementia*: 16.6 (15.3–18.4) *Control:* 18.3 (16.4–21.1) *p* = 0.008	**Total SB time was significantly greater in the impaired group** **Number of SB interruptions or number of 30 + min SB bouts did not significantly differ between groups** **Duration of SB bouts were significantly greater for impaired group**
Lu et al. (2018) [84] *Hong Kong* CS *Prevalence Pattern* *Study number in pinwheel* = *24*	*Healthy*: *n* = 271, Mage = 81.9 (3.5), % *F* = 38.2 *Low MoCA*: *n* = 252, Mage = 83.4 (4.0), % *F* = 47.6 *MCI: n* = 105, Mage = 83.6 (3.7), % *F* = 48.6 *AD*: *n* = 182, Mage = 80.8 (5.9), % *F* = 65.4 Population: mixed	**Device** (Actigraph)	﻿**Global Cognitive Function** (Hong Kong version of MoCA)	Model 1: age, gender, wear time Model 2: age, gender, wear time, years of education, BMI, unusual gait speed, living status, disease burden	**Time in SB (min/day):** Controls = 546.7 Low MoCA = 534.1 MCI = 516.9 AD = 601.2 **Average SB bout length:** *Controls:* 6.6 (0.2)* *Low MoCA:* 6.5 (0.2)* *MCI:* 6.3 (0.3)* *AD:* 7.9 (0.2) **# of SB bouts > 30 min = ** *Controls:* 3.3 (0.1)* *Low MoCA*: 3.3 (0.1)* *MCI:* 3.5 (0.2)* *AD:* 4.1 (0.1) *p < 0.05 compared to the AD group	**The most impaired group (AD) spent significantly more time in SB** **The most impaired group (AD) had significantly more 30 + min SB bouts**
Marmeleria et al. (2017) [85] *Portugal Prevalence* *Study number in pinwheel* = *N/A*	*Cognitively impaired:* *n* = 48, Mage = 83.9 (7.7), %*F* = 73, SB time = 604 MMSE = 14.9 (4.9) *Healthy: n* = 22, Mage = 82.2 (8.8), %*F* = 55, SB time = 601 MMSE = 25.8 (2.2.) Population: mixed	**Device** (Actigraph)	**Motor Skills and Construction** (Dear-Leiwald Reaction task)	age, gender, and accelerometer wear time	*Sedentary time* *p* > 0.05 *Reaction time* *p* > 0.05	**No significant difference in total SB time between the two groups**
Stubbs et al. (2017) [56] *Taiwan CS Prevalence* *Study number in pinwheel* = *30*	*Schizophrenia* *n* = 199, Mage = 44.0 (9.9) % *F* = 38.7, SB time = 581 *Controls* *n* = 60, Mage = 41.9 (9.6) % *F* = 43.3, SB time = 336 Population: mixed	**Device** (ActiGraph)	**Processing Speed**, (Cognitrone test (COG)) **Motor Skills and Construction** (Reaction Test, Grooved Pegboard Test (GPT))	Model 1: age, sex, education, weight status, smoking, alcohol consumption, medications, PANSS, MetS Model 2: age, sex, education, weight status, smoking, alcohol consumption, medications, PANSS, MetS, Physical activity energy expenditure	***p* < 0.001**	**Total SB time was significantly greater in the impaired group**
Suzuki et al. (2020) [57] *Japan CS Prevalence* *Study number in pinwheel* = *48*	*Males* *n* = 68, Mage = 88.0 (1.0), %*F* = 0, SB time = 855 *Females* *n* = 68, Mage = 88.0 (0.9), %*F* = 100, Sb time = 798 Population: mixed	**Device** (Actigraph)	**Global Cognitive Function** (ACE-III) Score of ≤ 88 indicating cognitive impairment	Single Factor Model: device wear time, age, education and the Center for Epidemiologic Studies Depression Scale Partition Model: All time units spent performing any of the activity categories and covariates were entered into the same model, and the independent effects of each behavioral variable were examined	**SB time (min), SD** **Males** *Cognitive Decline Group (n* = *54)* 859.1 (149.2) *Cognitive Maintain Group (n* = *14):* 837.4 (130.3) *p* = 0.363 **Females** *Cognitive Decline Group (n* = *50):* 788.6 (150.0) *Cognitive Maintain Group (n* = *18):* 824.8 (116.0) *p* = 0.357	**No significant difference in total SB time between the two groups**
van Alphen et al. (2016) [45] *Netherlands Prevalence* *Study number in pinwheel* = *N/A*	*Institutionalized dementia patients* *n* = 83, age: 83.0 (7.6), % *F* = 79.5 MMSE: 15.5 ± 6.5 *Community dwelling dementia patients* *n* = 37, Mage = 77.3 (5.6) % *F* = 40.5 MMSE = 20.8 (4.8) *Healthy older adults* *n* = 26, Mage = 79.5 (5.6) % *F* = 50 MMSE: 28.2 ± 1.6 Population: mixed	**Device** (Actigraph)	**Cognitive status** (MMSE)	Age, and cognitive state (MMSE)	SB time was different for the 3 groups (*F*(2,143) = 9.891, *p* < .001) ***institutionalized dementia patients:*** *SB h/day* = *17.30 (3.24)* ***community dwelling dementia patients:*** *SB h/day* = *15.83 (2.72)* ***healthy control:*** *SB h/day* = *14.54 (1.92)*	**Total SB time was significantly different in the three groups, with the greatest total SB time in the most impaired group**
Vancampfort et al. (2018) [52] *China, Ghana, India, Mexico, Russia, and South Africa* *CS* *Study number in pinwheel* = *31*	*Whole sample:* *n* = 32,715, Mage = 62.1 (15.6) % *F* = 50.1 *MCI* *n* = 4082, Mage = 64.4 (17.0) % *F* = 55.1, SB time = 262 Population: mixed	**Self-report** (Global physical activity questionnaire)	**Global Cognitive Function** (Based on the recommendations of the National Institute on Aging-Alzheimer’s Association)	Sex, age, years of education, wealth, depression, obesity, number of chronic conditions, low PA, country	***Sedentary* < *4 h/day, prevalence of MCI* = *13.5%*** ***Sedentary* ≥ 11 h/day, prevalence of MCI = 21.3%**	**The prevalence of MCI increased with increasing hours per day spent sedentary**
Varma et al. (2017) [86] *USA Prevalence* *Study number in pinwheel* = *N/A*	*Mild Alzheimer’s Disease* *n* = 36, Mage = 73.5 (7.9), % *F* = 28 SB time = 585 *Controls* *n* = 53, Mage = 73.2, %*F* = 70 SB time = 518 Population: mixed	**Device** (Actigraph)	**Cognitive Status** (clinical dementia rating scale scores) 0 (normal) 0.5 (very mild) 1 (mild)	Cardiorespiratory capacity, body mass index, mobility impairment, age, sex, and race	**Difference (Mild AD—Control)** sedentary minutes: − 5.738 (SE = 26.691) *p* = 0.830	**Total SB was significantly higher in the impaired group**
Watts et al. (2018) [54] *USA CS Prevalence Pattern* *Study number in pinwheel* = *35*	*Mild AD* *n* = 47, Mage = 73.1 (8.0) % *F* = 34. SB time = 584 *Controls* *n* = 53, Mage = 73.2 (6.5) % *F* = 69, SB time = 556.8 Population: mixed	**Device** (activPAL)	**Global Cognitive Function** (MMSE) **Processing Speed** (WAIS-DSST, block design, digits forward, animal fluency, vegetable fluency, TMT A) **Executive Function** (Stroop) **Cognitive Flexibility** (Letter number sequencing, TMT B) **Working Memory** (Letter number sequencing, digits backwards, TMT B) **Episodic Memory** (logical memory immediate, logical memory delayed) **Mild AD diagnosis:** = 0.5 (very mild) or 1 (mild) **Controls:** = 0 (no dementia)	None	**Mean SB time, (SD)** *p* = 0.52 *Mild AD activPAL:* 584.4 (91.2) *Controls activPAL:* 568.2 (130.8) **# interruptions, (SD)** *p* = 0.06 *Mild AD activPAL:* 42.28 (13.43) *Controls activPAL:* 47.52 (12.01) **30 + min bouts** *p* = 0.07 *Mild AD activPAL:* 5.49 (1.35) *Controls activPAL:* 4.91 (1.57)	**No significant difference in total SB time between the two groups** **Number of SB interruptions were significantly greater for the control group** **Number of 30 + min bouts per did not significantly differ between groups**

*AD* Alzheimer’s Disease, *ACE* Addenbrooke’s Cognitive Examination, *CVLT* California Verbal Learning Test, *DSST* Digit Symbol Substitution Task, *MMSE* (Mini-Mental State Examination), *Mage* Mean Age, *MCI* Mild Cognitive Impairment, *min* Minute, *MoCA* Montreal Cognitive Assessment, *PA* Physical Activity, *SB* Sedentary Behaviour, *SDMT* Symbol Digit Modalities Test, *TMT A* Trail Making Test A, *TMT B* Trail Making Test B, *WAIS* Wechsler Adult Intelligence Scale

**Table 4:** Summary and characteristics of experimental studies reporting an association of sedentary behaviour with cognitive function

Authors (year) *Country* *Study design* *Pinwheel number*	Participants Mean age (Mage) % F (female) SB time (min)	Design/intervention	Device or self-report (measure of sedentary behaviour)	Domain (outcome measure)	Covariates adjusted for	Results/conclusions
Duviver et al. (2017) [58] *Netherlands Experimental* *Study number in pinwheel* = *41*	*n* = 24, Mage = 64 (7) %*F* = 46 Population: non-clinical	*Randomized cross-over* *2 Conditions: 4 days each* **Condition 1:** restrict walking and standing to ≤ 1 h/day each, spending the remainder of the waking day sitting **Condition 2:** substitute at least 7 h/day of sitting with ≥ 4 h of self-perceived light walking and ≥ 3 h of standing; and to interrupt sitting preferably every 30 min with standing/walking bouts	**Device** (activPAL)	**Processing Speed** (TMT) **Working Memory** (TMT) **Cognitive Flexibility** (TMT) **Executive Function** (Attention Network test) **Episodic Memory** (immediate and delayed verbal memory)	Sex	No significant differences in cognitive outcomes between activity regiments
Edwardson et al. (2018) [59] *England Experimental* *Study number in pinwheel* = *42*	*Intervention* *n* = 77, Mage = 41.7 (11.0), %*F* = 73, SB time = 581 *Control* *n* = 69, Mage = 40.8 (11.3), %*F* = 87, SB time = 584.4 Population: non-clinical	*Cluster two arm randomized controlled trial* *3–12 months* Intervention group: multicomponent intervention (height adjustable desks, seminars, targets, feedback, posters, action planning, goal setting, self-monitoring and promt tool and coaching sessions) Control group: continued with usual practice	**Device** (activPAL)	**Processing Speed** (DDST) **Executive function** (Stroop) **Episodic Memory** (HVLT)	Baseline value, office size, and Average activPAL wear time, and average activPAL waking wear hours	A significant difference between groups (in favour of the intervention group) was found in **occupational sitting time** at 3, 6 and 12 months (− 83.28 min/workday, 95% confidence interval − 116.57 to − 49.98, *P* = 0.001) Differences between groups (in favour of the intervention group compared with control) were observed for **daily sitting time** at six months (− 59.32 min/day, − 88.40 to − 30.25, *p* < 0.001) and 12 months (− 82.39 min/day, − 114.54 to − 50.26, *P* = 0.001) There were differences between groups in reaction times at 3, 6, and 12 months for the congruent level of the Stroop Colour-Word Test and in proportion of correct hits at the incongruent level, all in favour of the intervention group compared with control
Ezeugwu et al. (2018) [60] *Canada* *Experimental* *Study number in pinwheel* = *9*	*n* = 34, Mage = 64.6 (12.5) % *F* = 44.0 Population: clinical	*Single group intervention study* 1-week baseline 8-week intervention 8-week follow-up Aimed to interrupt and replace sedentary time with upright activities at home and in the community	**Device** (activPAL)	**Global Cognitive Function** (MoCA)	Age and sex	Sedentary time decreased by 54.213.7 min per day (*p* < .01) at postintervention and 26.814.0 min per day (PZ.07) at follow-up, relative to baseline Significant improvement in cognition over-time
Falck et al. (2018) [70] *Canada* *Experimental* *Study number in pinwheel* = *11*	*I-INT* *n* = 30, Mage = 61.7 (9.4) % *F* = 73.3, SB time 682 *D-INT* *n* = 31, Mage = 62.6 (8.5) % *F* = 90.3, SB time = 703 Population: non-clinical	*Secondary analysis of a 6-month randomized controlled trial* *2 groups* - Immediate intervention - Delayed intervention (control group; received same intervention as I-INT after a 2-month wait) Intervention: 1.5 h group education session & individual counselling to increase MVPA and decrease SB	**Device** (SenseWear Mini & FitBit flex)	**Episodic Memory** (Picture sequence) **Working Memory** (List sorting)	Baseline cognitive scores	There were no statistically significant relationships between changes in SB and changes in either picture sequence memory (*B* = −0.01; 95% CI [− 0.09, 0.07]) or list-sorting memory (*B* = 0.00; 95% CI [− 0.09, 0.10])
Maasakkers et al. (2020) [73] *Netherlands Experimental* *Study number in pinwheel* = *44*	*n* = 22, Mage = 78 (5.3) %*F* = 41, SB time = 618 Population = non-clinical	*Randomized cross-over* Condition 1: 3 h of sitting Condition 2: 3 h of sitting interrupted every 30 min with 2 min of walking	**Device** (activPAL) & lab-supervised	**Executive Function** (Attentional performance battery) **Working Memory** (Attentional performance battery)	Corrected for the order of the first measurement condition	No short-term differences were observed in cognitive performance across time or between conditions
Marusic et al. (2020) [71] *USA* *Experimental* *Study number in pinwheel* = *46*	*n* = 12, Mage = 71.1 (3.8) %*F* = 25 Population: clinical	*Randomized cross-over* Condition 1 (3-h): “baseline”- participants remained seated throughout the period except for the rare break Condition 2 (4-h): “static standing”-participants were asked to stand behind the table Condition 3 (4-h): “dynamic standing”- participants stood behind the same table but received periodic cues to induce weight-shifting steps	**Device** (Actigraph)	**Working Memory** (TMT) **Cognitive Flexibility** (TMT) **Executive Function** (Stroop)	Not stated	Significant beneficial effects of standing conditions for Stroop and some TMT sub-tests
Wanders et al. (2020) [72] *Netherlands Experimental* *Study number in pinwheel* = *51*	*n* = 24, Mage = 60 (8.0), %*F* = 79, SB time = 612 Population: Healthy *Condition 1:* 4-h uninterrupted sitting (SIT) *Condition 2:* Sitting interrupted by PA breaks (5-min cycling every 30 min) (ACT)	*Randomized cross-over* 4-h of uninterrupted sitting vs. 4-h of interrupted sitting (5-min cycling every 30 min)	**Device** (activPAL)	**Motor Skills and Construction** (Reaction time from the Computer-based Test of Attentional Performance) **Working Memory** (Computer-based Test of Attentional Performance) **Executive Function** (Flexibility Score from the Computer-based Test of Attentional Performance)	Not stated	PA breaks had no significant effects on the cognitive outcomes
Wheeler et al. (2019) [62] *Australia Experimental* *Study number in pinwheel* = *36*	*n* = 67, Mage = 67 (7.0) % *F* = 52.2, SB time = n/a Population: non-clinical	*Randomized cross-over* Condition 1: uninterrupted sitting (8 h, control) Condition 2: sitting (1 h), moderate-intensity walking (30 min), uninterrupted sitting (6.5 h) Condition 3: sitting (1 h), moderate-intensity walking (30 min), sitting interrupted every 30 min with 3 min of light-intensity walking (6.5 h)	**Lab-supervised**	**Executive Function** (Groton Maze Learning Test) **Processing Speed** (Detection Test, Identification Test) **Episodic Memory** (One Card Learning Test) **Working Memory** (n-back)	Age, sex, waist circumference, treatment order, testing site, baseline values, years of education	A morning bout of moderate-intensity exercise improved executive function over an 8-h period in older adults, relative to prolonged sitting When exercise was combined with light-intensity breaks in sitting, working memory but not executive function was improved, relative to prolonged sitting

*ACE* Addenbrooke’s Cognitive Examination, *AF* Animal Fluency, *AOS* Automated Operation Span, *BVMT* Brief Visuospatial Memory Test, *CVLT* California Verbal Learning Test, *COG* Cognitrone Test, *DSB* Digit Span Backwards, *DSST* Digit Symbol Substitution Task, *FLA* Flanker or Eriksen Flanker Test, *GPT* Grooved Pegboard Test, *HVLT* Hopkins Verbal Learning Test, *Mage* Mean Age, *MMSE* Mini-Mental State Examination, *MoCA* Montreal Cognitive Assessment, *NR* Not reported, *PASAT* Paced Auditory Serial Addition Test, *RT* Reaction Time, *SB* Sedentary Behaviour, *SDMT* Symbol Digit Modalities Test, *TMT A* Trail Making Test A, *TMT B* Trail Making Test B, *VF* Verbal Fluency, *WAIS* Wecshler Adult Intelligence Scale, *WCST* Wisconsin Card Sorting Test

### Quality Assessment

Two review authors assessed the quality of each paper independently using the study quality checklist *QualSyst* proposed by Kmet et al. (2004), allowing assessment of both experimental and quasi-experimental studies [31]. Studies were scored (0—No, 1—Partial, 2—Yes) on 14 criteria. Aspects covered included the quality of study design, confounders, blinding, selection bias and misclassification bias. The sum of all scores was then divided by the highest possible score for each study. Any discrepancies between the two authors’ assessment were further discussed until a quality percentage score was agreed upon. A quality score of ≥ 75% indicates strong quality, a score between 55 and 75% moderate quality, and a score < 55% weak quality.

### Data Synthesis and Analysis

#### Systematic Review

First, regardless of the statistical procedure used, all studies that reported an association (cross-sectional, longitudinal and experimental studies) of sedentary behaviour with cognitive function were synthesized into a pinwheel based on the method of sedentary behaviour measurement and the cognitive domain that was assessed. Method of measurement was separated into three categories (1) self-report; (2) activPAL™ or (3) other device (i.e., Actigraph). The two device-based measures were separated due to activPAL being the gold standard for measuring sedentary time, as they can differentiate sitting from standing. Cognitive function was separated into the seven various cognitive domain categories stated previously. Second, studies were synthesized based on associations reported using sedentary behaviour as a categorical variable (i.e., high versus low groupings). Third, studies that reported on the pattern of sedentary time accumulation (i.e., 30 + min bouts or number of interruptions) and its association with cognitive function. Fourth and finally, studies comparing prevalence of sedentary behaviour between healthy versus cognitively impaired populations.

#### Meta-analysis

Studies that were of high quality (i.e., scoring 75% or greater on the quality score outcome) were considered for inclusion in the meta-analysis. Studies that analysed cognitive function and sedentary behaviour as continuous variables were pooled into a random effects model meta-analysis using Comprehensive Meta-analysis Software (version 3). Random-effects models were chosen as heterogeneity was expected given the differences in study populations and procedures. Heterogeneity was determined by Cochran’s* Q* statistic and *I*^2^ values (values of 25, 50 and 75 were considered to indicate low, moderate and high heterogeneity, respectively) [32]. Planned sub-group and meta-regression analyses were conducted to examine the contribution of specific variables to heterogeneity. These were specified a priori and were as follows: measurement type (activPAL™, other device or self-report), outcome assessment (cognitive flexibility, episodic memory, executive function, global cognitive function, motor skills and construction, processing speed and working memory), physical activity (studies controlling for physical activity time), number of covariates (number of covariates used in the analysis), age (mean age of population), percent female (percent of the population reported as females) and sedentary time (sedentary time reported in hours). Sub-group analyses were employed when five or more studies were available that used the same design (i.e., cross-sectional), and reported a correlation, standardized or unstandardized regression coefficient [33]. Where *r* was not reported, we transformed standardized beta (*β*) values to *r* based on the formula described by Peterson et al. (2005) [*r* = *β* + 0.05*λ* [34]. Where unstandardized betas were reported, they were converted to standardized values with the following formula: [standardized beta = unstandardized beta *x* (SD of independent variable/SD of the dependent variable)]. If a study reported data for more than one model, the least adjusted model was used within the analysis to improve comparability between studies. If a study reported data for more than one outcome, each study was used as the unit of analysis in the overall model. Studies reporting prevalence of sedentary time between populations diagnosed with mild cognitive impairment or dementia and populations considered cognitively healthy were also analyzed in a random effect meta-analysis using means, sample sizes and p values. If a study was deemed unsuitable for the meta-analysis (i.e., units not comparable or missing key information) the authors were contacted in an attempt to obtain the necessary data. We had intended to also calculate the pooled mean effect of the longitudinal, experimental and studies reporting on patterns of sedentary behaviour time accumulation. However, this was not possible due to the low number and heterogeneity between the studies. Publication bias was assessed using a funnel plot of all included studies to allow for visual inspection of publication bias.

## Results

### Search Results/Study Characteristics

**Fig. 1:**
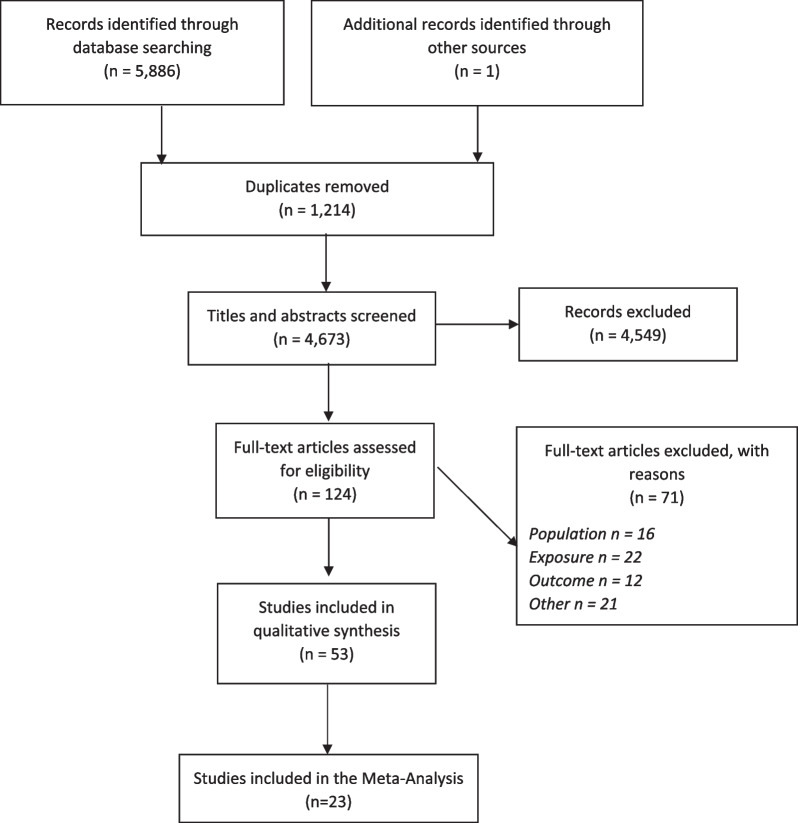
PRISMA flow diagram of the search and screening process in review of sedentary behaviour with cognitive function

The initial electronic database search identified 5886 papers with one additional paper found through forward searching. After duplicates were removed, 4673 papers remained for title and abstract screening. After the initial screening stage, 124 papers were identified for full text review. Following the full text review, 71 were excluded (see Additional file 3 for detailed reasons of exclusion) leaving 53 to meet the inclusion criteria (see Fig. 1 for the PRISMA flow diagram). Twenty-eight of the 53 studies were secondary data analyses; with 41 of the studies being published in 2017 or later. Tables 1, 2, 3 and 4 summarize the characteristics of the included studies, including participant characteristics, exposure measurement method, outcomes reported (task and corresponding domain), covariates used and the main outcomes of interest. Of the 53 studies included in this review, most were observational in nature. Forty-three studies employed a cross-sectional design; four of those reporting both cross-sectional and longitudinal data [35–38] and two studies reporting longitudinal data only [39, 40]. Three of the 43 cross-sectional studies also implemented isotemporal substitution models [41–43] and the remaining eight consisted of various experimental designs (i.e., randomized crossover). The average quality score was 83%, ranging from 59 to 100% (see Table 5).

**Table 5:** Quality assessment of included studies

References	1. Question/objective sufficiently described	2. Appropriate study design	3. Appropriate method/source of selection	4. Characteristics sufficiently described	5. Random allocation described	6. Blinding of investigators	7. Blinding of subjects	8. Outcome/exposure measures defined and robust	9. Appropriate sample size	10. Appropriate analytical methods	11. Estimate of variance reported	12. Controlled for confounding	13. Results reported sufficiently	14. Conclusions supported by results	15. Quality score (%)
Amagasta et al. [77]	1	2	2	2	n/a	n/a	n/a	1	2	2	2	2	2	2	91
Bojsen-Moller et al. [50]	2	2	2	2	n/a	n/a	n/a	1	2	2	2	2	2	2	95
Bollaert & Motl [68]	2	1	2	2	n/a	n/a	n/a	1	2	2	2	1	2	2	86
Burzynska et al. [69]	2	2	2	2	1	2	n/a	1	2	2	2	2	2	2	92
Cukic et al. [35]	1	2	2	1	n/a	n/a	n/a	2	2	2	2	2	2	2	91
Da Ronch et al. [46]	1	2	1	2	n/a	n/a	n/a	1	2	2	2	2	1	1	71
Duvivier et al. [58]	2	2	1	2	n/a	n/a	n/a	2	2	2	2	2	2	2	95
Edwardson et al. [59]	2	2	2	2	2	2	n/a	2	2	2	2	2	2	2	100
Ehlers et al. [41]	2	2	2	2	n/a	n/a	n/a	1	1	2	2	2	2	2	91
Ekblom et al. [90]	1	2	1	2	n/a	n/a	n/a	1	1	1	1	2	1	1	64
English et al. [74]	2	2	1	2	n/a	n/a	n/a	2	1	1	2	0	1	2	73
Ezeugwu et al. [75]	2	2	1	2	n/a	n/a	n/a	2	0	2	1	0	2	1	68
Ezeugwu et al. [60]	1	2	2	2	n/a	n/a	n/a	2	1	2	2	1	2	1	82
Falck et al. [55]	2	2	1	2	n/a	n/a	n/a	1	2	2	2	2	2	2	91
Falck et al. [70]	1	2	2	1	2	0	n/a	1	1	1	2	1	1	1	62
Fanning et al. [42]	1	2	2	2	n/a	n/a	n/a	1	2	2	2	2	2	2	91
García-Hermoso et al. [48]	2	2	2	2	n/a	n/a	n/a	1	2	2	2	2	2	2	95
Hartman et al. [83]	1	2	2	1	n/a	n/a	n/a	1	1	2	2	0	1	2	68
Hayes et al. [61]	2	1	1	2	n/a	n/a	n/a	1	0	2	2	2	1	2	73
Hubbard et al. [79]	2	2	1	2	n/a	n/a	n/a	1	2	1	0	0	2	1	64
Iso-Markku et al. [78]	1	2	2	2	n/a	n/a	n/a	1	2	2	1	2	1	2	82
Johnson et al. [65]	2	1	1	2	n/a	n/a	n/a	1	2	2	2	2	2	2	86
Kojima et al. [40]	1	1	2	2	n/a	n/a	n/a	1	1	1	2	1	1	2	68
Koolhaas et al. [37]	1	2	2	2	n/a	n/a	n/a	1	2	2	2	2	2	2	91
Ku et al. [36]	2	2	2	2	n/a	n/a	n/a	1	2	2	2	2	2	2	95
Lee et al. [39]	1	2	1	1	n/a	n/a	n/a	1	2	1	2	2	1	1	68
Leung et al. [76]	2	2	1	2	n/a	n/a	n/a	1	1	2	0	0	1	1	59
Lopes et al. [82]	2	2	2	2	n/a	n/a	n/a	1	2	2	2	0	2	1	82
Lu et al. [84]	1	2	1	2	n/a	n/a	n/a	1	2	2	2	2	2	1	82
Maasakkers et al. [38]	2	2	2	2	n/a	n/a	n/a	1	2	2	2	2	2	2	95
Maasakkers et al. [73]	2	2	2	2	1	0	n/a	1	2	2	2	1	2	2	81
Marinac et al. [64]	1	2	2	2	n/a	n/a	n/a	2	1	2	1	2	1	1	77
Marmeleria et al. [85]	2	1	2	2	n/a	n/a	n/a	1	1	2	2	2	2	2	86
Marusic et al. [71]	2	2	2	2	1	0	n/a	1	0	2	2	0	2	2	69
Olanrewaju et al. [80]	2	2	2	2	n/a	n/a	n/a	1	2	2	2	2	2	2	95
Rosenberg et al. [51]	2	1	1	2	n/a	n/a	n/a	1	2	2	2	2	2	2	86
Siddarth et al. [81]	2	2	2	2	n/a	n/a	n/a	1	1	2	2	1	1	2	82
Snethen et al. [44]	1	1	1	2	n/a	n/a	n/a	1	0	2	1	2	2	2	68
Stubbs et al. [56]	2	0	0	1	n/a	n/a	n/a	1	2	2	1	2	2	1	64
Suzuki et al. [57]	2	2	2	2	n/a	n/a	n/a	1	1	2	2	2	2	2	91
van Alphen et al. [45]	2	2	1	2	n/a	n/a	n/a	1	2	2	2	2	2	2	91
Vancampfort et al. [52]	2	2	2	2	n/a	n/a	n/a	1	2	2	0	2	2	1	82
Vance et al. [49]	1	1	1	2	n/a	n/a	n/a	1	2	2	0	0	2	1	59
Varma et al. [86]	2	2	2	2	n/a	n/a	n/a	1	2	2	2	2	2	2	95
Vasquez et al. [47]	2	2	2	2	n/a	n/a	n/a	1	2	2	2	2	2	1	91
Wanders et al. [72]	2	2	2	2	1	2	n/a	2	1	2	2	0	2	2	85
Wanigatunga et al. [53]	1	2	2	2	n/a	n/a	n/a	1	2	1	2	2	2	2	86
Watts et al. [54]	2	2	2	2	n/a	n/a	n/a	2	1	2	2	1	2	2	91
Wei et al. [43]	2	2	2	2	n/a	n/a	n/a	1	2	2	2	2	2	2	95
Wheeler [62]	2	2	2	2	2	2	2	2	1	2	2	2	2	2	96
Wu et al. [63]	2	2	2	2	n/a	n/a	n/a	1	1	2	2	2	2	2	91
Zhu [67]	2	2	1	2	n/a	n/a	n/a	1	2	1	1	2	1	2	77
Zlatar [66]	2	2	1	2	n/a	n/a	n/a	1	1	2	2	2	2	1	82
Total average															83

#### Population

The total sample size from the 53 studies was 83,137 middle-aged and older adults (study population sizes ranged from 12 to 32,715 (median of 150) with mean ages ranging from 40.8 to 88.0 years old. Studies were mainly conducted in the USA (*n* = 20), but other countries included were Canada (*n* = 5), Australia (*n* = 4), Japan (*n* = 5), Netherlands (*n* = 6), Sweden (*n* = 2), Taiwan (*n* = 2), Brazil (*n* = 1), Chile (*n* = 1), China (*n* = 2), England (*n* = 1), Finland (*n* = 1), Germany (*n* = 1), Ghana (*n* = 1), Hong Kong (*n* = 1), India (*n* = 1), Ireland (*n* = 1), Italy (*n* = 1), Mexico (*n* = 1), Portugal (*n* = 1), Russia (*n* = 1), Scotland (*n* = 1), Singapore (*n* = 1), South Africa (*n* = 1) and Switzerland (*n* = 1).

#### Exposure (Sedentary Time)

Tables 1, 2, 3 and 4 describe and summarize the method of sedentary behaviour measurement for each study. The majority of the studies (*n* = 44) used a device (i.e., accelerometer, inclinometer) to measure sedentary behaviour. Eight various devices were used, and only 10 of the 44 device-based studies used an activPAL™. The reported device measured sedentary behaviour time ranged from 405 min per day [44] to 1038 min per day [45]. Three of the studies using a device also used a self-report measure. Eight studies used only a self-reported measure. Five different self-report measures were used, and two studies [46, 47] did not specify their measurement tool. Self-reported sedentary behaviour time ranged from 225 min per day [48] to 803 min per day [49].

#### Outcome (Cognitive Function)

Tables 1, 2, 3 and 4 include all measures used within each study and its corresponding cognitive domain. Processing speed and episodic memory were assessed with 16 and 15 different measures respectively. Nine different measures were used to assess global cognitive function. Ten different measures were used to assess executive function, seven for cognitive flexibility and eleven for working memory. Lastly, five different measures were used to assess motor skills and construction.

### Association of Total Sedentary Time with Cognitive Function

**Fig. 2:**
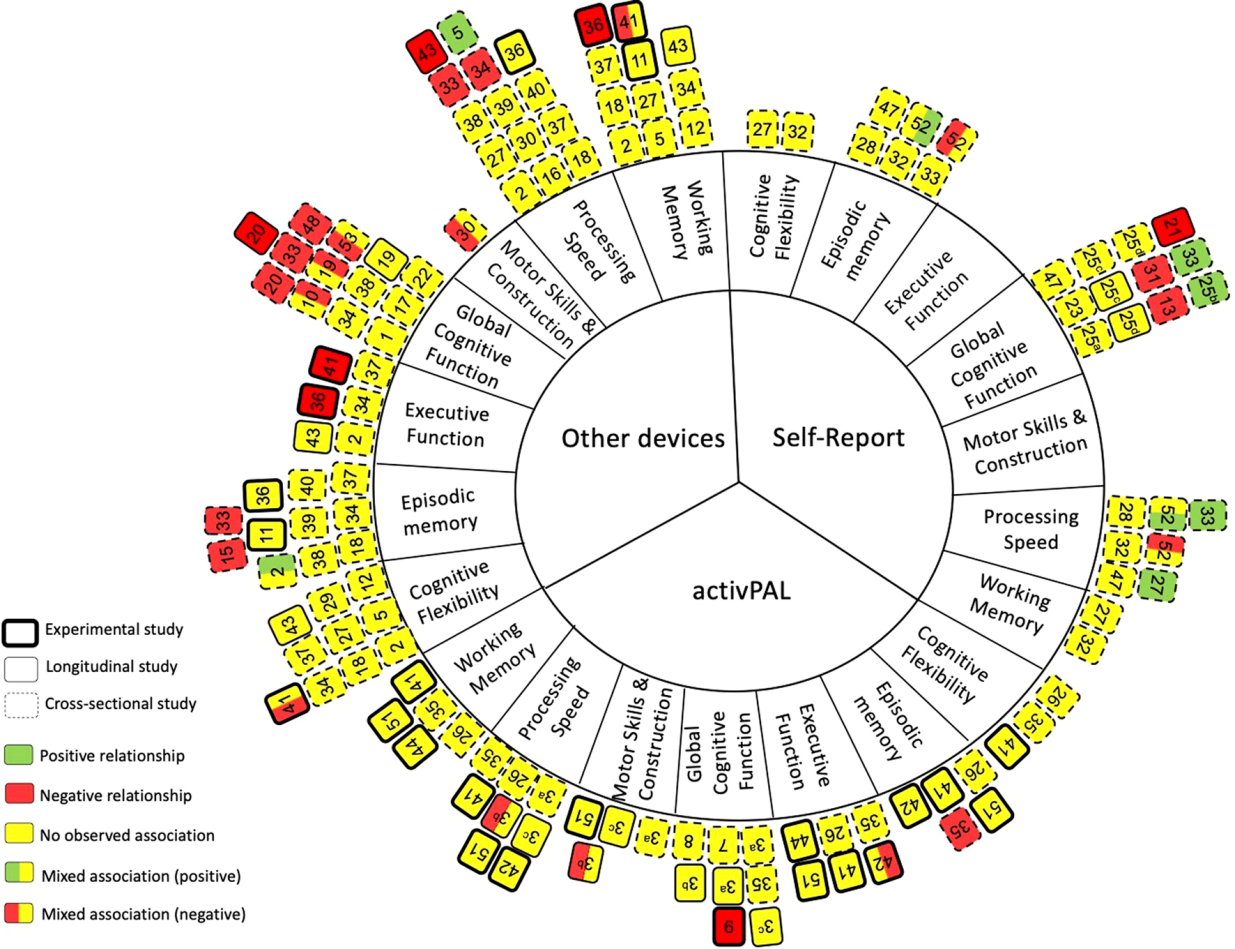
Forest plot of all studies reporting on an association of sedentary behaviour with cognitive function based on method of measurement (device or self-report) and cognitive domain

Studies assessing the association of sedentary time with cognitive function are summarized in Tables 1, 2, 3 and 4 and illustrated in Fig. 2. Overall, most studies report no association, with some reporting that more sedentary time was associated with worse cognitive function and the fewest studies reporting the more sedentary time was associated with better cognitive function. We can also observe that experimental studies in the area are lacking, with mixed associations found from longitudinal studies.

#### Association of Total Sedentary Time with Cognitive Function Based on Measurement Type

Figure 2 illustrates that six studies showed positive associations [38, 41, 43, 47, 50, 51] (i.e., more sitting resulting in better cognitive function), with most of these studies (*n* = 4) using self-reported measures of total sedentary behaviour time [38, 43, 47, 51]. Figure 2 also shows that 20 studies found negative associations [35–37, 39, 40, 43, 47, 48, 52–63] (i.e., more sitting resulting in worse cognitive function), with most of these studies (*n* = 16) using a device to measure total sedentary behaviour time [35–37, 40, 47, 53–63].

#### Association of Total Sedentary Time with Cognitive Function Based on Cognitive Domain

Associations of sedentary time with cognitive function, based on cognitive domain are summarized in Tables 1, 2, 3 and 4 and illustrated in Fig. 2. For studies using a device for measurement of sedentary behaviour time, there were 12 studies assessing cognitive flexibility [40–42, 44, 50, 51, 53, 54, 58, 64–66], 16 studies for episodic memory [47, 50, 53, 54, 58, 59, 61, 62, 65–72], 11 studies for executive function [40, 50, 53, 54, 58, 59, 62, 66, 71–73], 16 studies for global cognitive function [35–37, 47, 53–55, 57, 60, 63, 67, 74–78], three studies reporting on the domain of motor skills and construction [35, 56, 72], 20 studies for processing speed [35, 40, 41, 41, 47, 50, 51, 53, 54, 56, 58, 59, 62, 65–69, 71, 72, 79] and 15 for working memory [40–42, 50, 51, 53, 54, 58, 62, 64–66, 70, 72, 73]. For studies using self-report to measure sedentary behaviour time, there were two studies for cognitive flexibility [49, 51], five studies for episodic memory [43, 47, 49, 80, 81], seven for global cognitive function [38, 39, 47, 48, 52, 80, 82], six for processing speed [43, 47, 49, 51, 80, 81] and two for working memory [49, 51]. Overall, results of the studies were mixed, with no consistent associations found for any of the domains. Figure 2 demonstrates that the relationship of sedentary time with some cognitive domains remains unexplored (i.e., domain of motor skills and construction and executive function for studies using self-report to measure sedentary behaviour time).

#### High Versus Low Sedentary Time

More detailed results of the studies that used categories of sedentary time can be found in Table 1. One study grouped sedentary time into two groups (i.e., high versus low) [52]. They report that those in the high sedentary time group had significantly higher odds for mild cognitive impairment. Three studies [36, 53, 82] separated sedentary time into tertiles. One study [82] reported the people in the highest tertile of sedentary time had the highest cognitive function while the other two [36, 53] found the opposite (i.e., participants in the highest tertile of sedentary time had the worst cognitive function). One study used quartiles to group sedentary time, based on level of physical activity. They reported no associations for time spent in sedentary behaviour with any of their cognitive measures [67].

#### Associations Based on Pattern of Accumulation

Table 2 describes the 11 studies that assessed the association of various patterns of sedentary time accumulation with cognitive function [35, 53–55, 64, 68, 74–76, 83, 84]. Six studies investigated whether more prolonged bouts of sedentary time (30 + min) were associated with cognitive function [53, 55, 64, 68, 74, 76]. Four studies reported no association [53, 68, 74, 76] and two studies reported a significant association [55, 64] for more prolonged sedentary bouts with worse cognitive function. Five studies reported on sedentary behaviour pattern of accumulation (i.e., interruptions in sitting time via sit to stand transitions, duration of sedentary bouts or number of sedentary behaviour bouts) and cognitive function, and all five observed no association [35, 64, 68, 75, 76].

#### Prevalence (Cognitively Impaired vs. Healthy)

Table 3 describes the 12 studies that assessed the prevalence of sedentary time between populations with cognitive impairment or dementia and cognitively healthy populations [45, 46, 52, 54–57, 68, 77, 83–86]. Eight studies reported the cognitively impaired population spending more time sedentary [45, 46, 52, 56, 68, 83, 84, 86] while five studies reported no significant difference [54, 55, 57, 77, 85]. Five studies reported if prolonged bouts of sedentary time differed between cognitively impaired and non-impaired populations [54, 55, 68, 83, 84]. Three studies reported the number of prolonged bouts did not differ between cognitively impaired and non-impaired populations [54, 83, 84] and two studies reported the number of prolonged bouts did significantly differ [55, 68]. For pattern of sedentary behaviour time accumulation, three studies reported no difference between the two groups [54, 68, 83].

#### Experimental Studies

Figure 2 illustrates the eight studies that utilized an experimental design and Table 4 describes them in detail. The experimental periods ranged from 3 h to 12 months. Overall, the shorter experimental protocols (i.e., 3 h to four days) showed less consistent findings than the longer-term protocols (i.e., eight weeks to 12 months). For example, two of the three studies that were eight weeks or longer showed a positive effect of reducing sedentary behaviour on cognitive functioning while only one of the five shorter-term studies (i.e., four days or less) showed any benefit.

### Meta-analysis Results

#### Total Sedentary Time and Cognitive Function

**Fig. 3:**
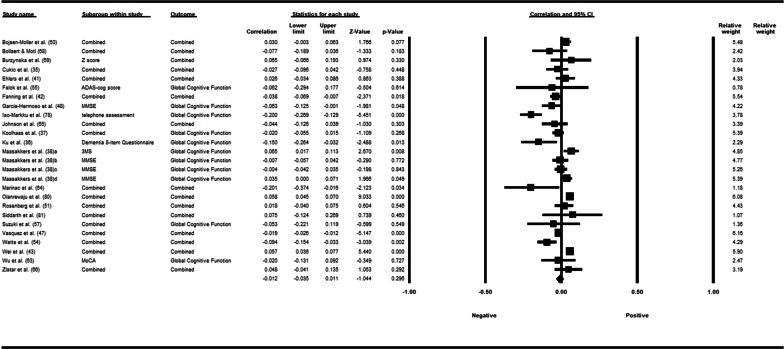
Forest plot of all eligible studies for the meta-analysis reporting on the association of sedentary behaviour with cognitive function using a random-effects model. *ACE-III* Addenbrooke’s Cognitive Examination, *ADAS-cog* Alzheimer’s Disease Assessment Scale—Cognitive, *MMSE* Mini Mental State Examination, *3MS* Modified Mini Mental State Examination, *MoCA* Montreal Cognitive Assessment. Note a is the SALSA cohort, b is the PATH cohort, c is the SGS cohort, and d is the SLAS2 cohort reported within one paper

**Fig. 4:**
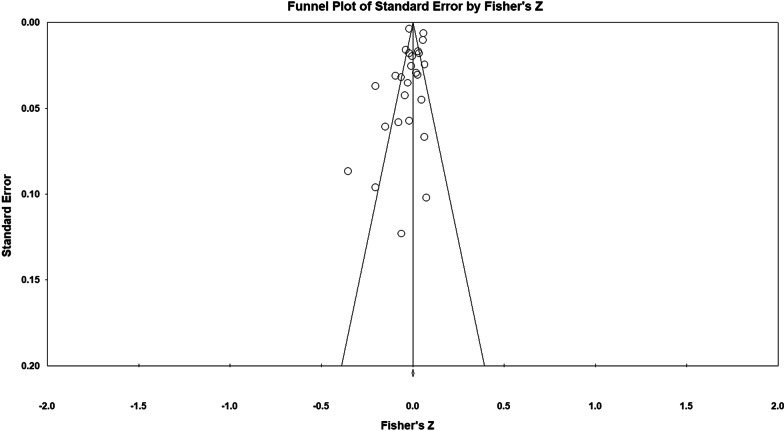
Funnel plot for all eligible studies in the meta-analysis reporting on the association of sedentary behaviour with cognitive function using a random-effects model

Twenty-three studies including 41,334 participants were pooled and showed a non-significant association of higher sedentary time with worse cognitive function (Fig. 3: *r* = −0.012 [95% CI − 0.035, 0.011], *p* = 0.296). Heterogeneity between the studies was statistically significant (*Q* = 219.694, *df* = 25, *p* < 0.001) and large in magnitude (*I*^2^ = 89%). Visual assessment of the funnel plot (Fig. 4) suggests no notable asymmetry.

#### Meta-regression

The results of the a priori subgroup meta-regression analyses to explore heterogeneity are shown in Table 6. Measurement type and sedentary time were significant (*p* < 0.05).

**Table 6:** Meta-regression main results for random effects

Variable	Number of studies	Comparison groups	*β*	95% CI	*p* value	*R-*squared
Measurement type^a^	31	n/a	0.075	[0.038, 0.111]	** < 0.001**	0.63
Physical activity^b^	27	n/a	− 0.062	[− 0.135, − 0.012]	0.102	0.10
Number of covariates	27	n/a	− 0.006	[− 0.173, 0.006]	0.337	0.00
Age	27	n/a	− 0.003	[− 0.006, 0.004]	0.092	0.05
% Female	27	n/a	− 0.001	[− 0.001, 0.004]	0.327	0.00
Sedentary time (h)	27	n/a	− 0.017	[− 0.029, − 0.004]	**0.009**	0.28

^a^Device or self-report

^b^Studies that included physical activity as a covariate. Bold indicate that the significance of *p* values < 0.05

#### Subgroup Analyses of Device-Based Studies

**Fig. 5:**
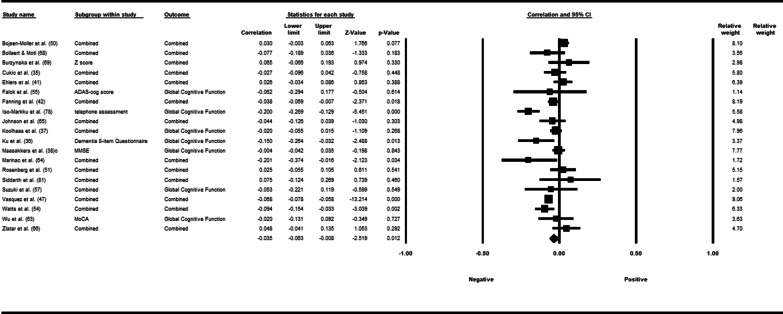
Forest plot of all eligible studies for the meta-analysis that used a device as the method a measurement for sedentary behaviour reporting on the association of sedentary behaviour with cognitive function using a random-effects model. c is the SGS cohort. *ADAS-cog* Alzheimer’s Disease Assessment Scale—Cognitive, *MMSE* Mini Mental State Examination, *MoCA* Montreal Cognitive Assessment

Subgroup analyses of studies using a device to measure sedentary time suggested that more sedentary time was significantly associated with worse cognitive function (Fig. 5; *r* = −0.035 [95% CI − 0.063, − 0.008], *p* = 0.012). Heterogeneity between the studies was statistically significant (*Q* = 87.629, *df* = 19, *p* < 0.001) and large in magnitude (*I*^2^ = 78%).

#### Subgroup Analyses of Cognitive Domains from Device-Based Studies

**Fig. 6:**
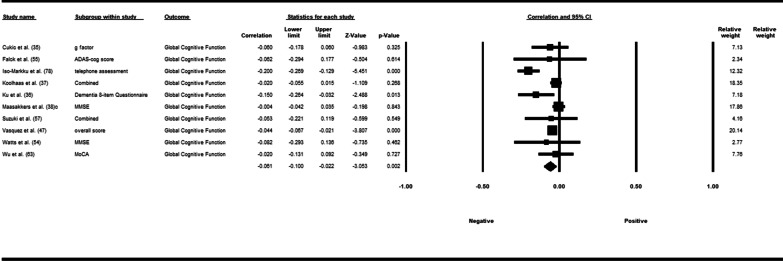
Forest plot of all eligible studies for the meta-analysis that used a device as the method a measurement for sedentary behaviour reporting on the association of sedentary behaviour with the cognitive domain of global cognitive function using a random-effects model. c is the SGS cohort. *ADAS-cog* Alzheimer’s Disease Assessment Scale—Cognitive, *MMSE* Mini Mental State Examination, *MoCA* Montreal Cognitive Assessment

**Fig. 7:**
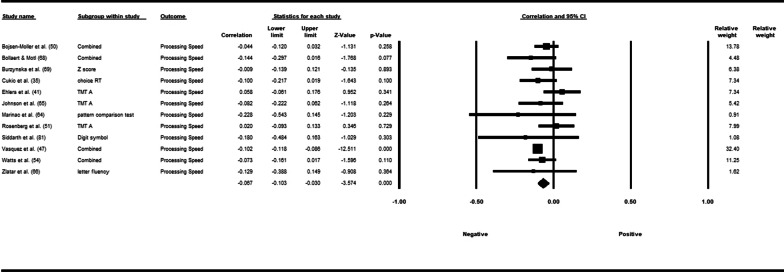
Forest plot of all eligible studies for the meta-analysis that used a device as the method a measurement for sedentary behaviour reporting on the association of sedentary behaviour with the cognitive domain of processing speed using a random-effects model. *RT* reaction time; *TMT A* Trail Making Test A

**Fig. 8:**
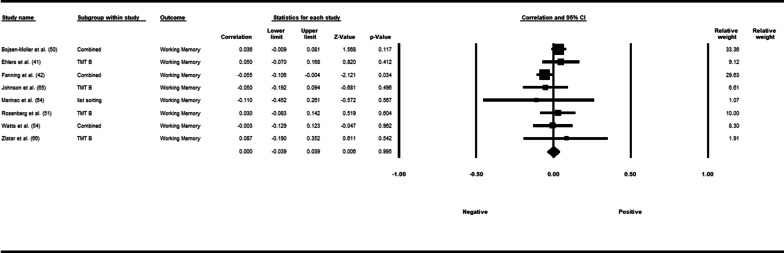
Forest plot of all eligible studies for the meta-analysis that used a device as the method a measurement for sedentary behaviour reporting on the association of sedentary behaviour with the cognitive domain of working memory using a random-effects model. *TMT B* Trail Making Test B

**Fig. 9:**
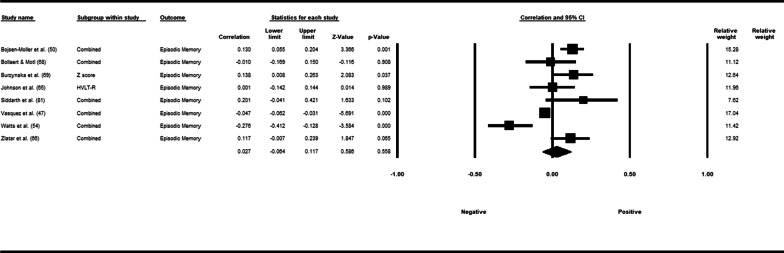
Forest plot of all eligible studies for the meta-analysis that used a device as the method a measurement for sedentary behaviour reporting on the association of sedentary behaviour with the cognitive domain of episodic memory using a random-effects model. *HVLT-R* Hopkins Verbal Learning Test-Revised

**Fig. 10:**
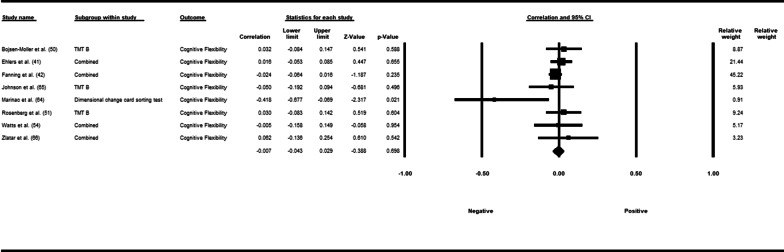
Forest plot of all eligible studies for the meta-analysis that used a device as the method a measurement for sedentary behaviour reporting on the association of sedentary behaviour with the cognitive domain of cognitive flexibility using a random-effects model. *TMT B* Trail Making Test B

There were sufficient studies to meta-analyse the relationship of device-based sedentary time with five of the seven domains of cognitive function. Global cognitive function and processing speed showed significant negative associations for sedentary time with cognitive function (Fig. 6; *r* = −0.061 [95% CI − 0.100, − 0.022], *p* = 0.002; *Q* = 27.597, *df* = 9, *p* < 0.01, *I*^2^ = 67% and Fig. 7; *r* = −0.067, [95% CI − 0.103, − 0.030], *p* < 0.001; *Q* = 15.858, *df* = 11, *p* = 0.146, *I*^2^ = 31% respectively). No associations were found for the domains of working memory (Fig. 8; *r* = 0.000 [95% CI − 0.039, 0.039], *p* = 0.995; *Q* = 9.065, *df* = 7, *p* = 0.248, *I*^2^ = 23%), episodic memory (Fig. 9; *r* = 0.027 [95% CI − 0.064, 0.117], *p* = 0.558; *Q* = 46.583, *df* = 7, *p* < 0.001, *I*^2^ = 85%) or cognitive flexibility (Fig. 10; *r* = −0.007 [95% CI − 0.043, 0.029], *p* = 0.698; *Q* = 7.929, *df* = 7, *p* = 0.339, *I*^2^ = 12%).

#### Subgroup Analyses of Self-Report-Based Studies

**Fig. 11:**
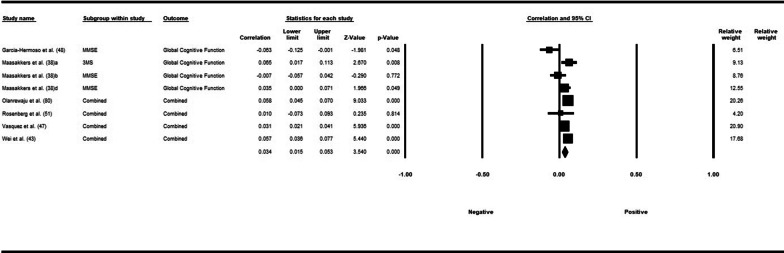
Forest plot of all eligible studies for the meta-analysis that used self-report as the method a measurement for sedentary behaviour reporting on the association of sedentary behaviour with cognitive function using a random-effects model. *MMSE* Mini Mental State Examination, *3MS* Modified Mini Mental State Examination. Note a is the SALSA cohort, b is the PATH cohort, and d is the SLAS2 cohort reported within one paper

**Fig. 12:**
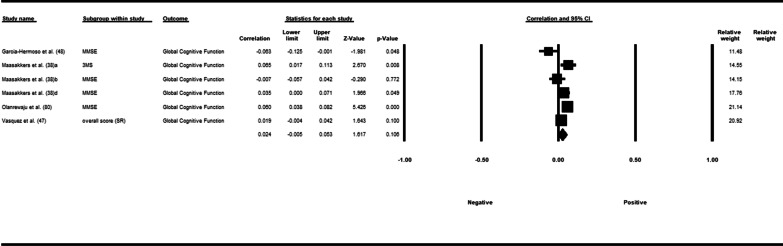
Forest plot of all eligible studies for the meta-analysis that used self-report as the method a measurement for sedentary behaviour reporting on the association of sedentary behaviour with the cognitive domain of global. *MMSE* Mini Mental State Examination, *3MS* Modified Mini Mental State Examination, *SR* self-report. Note a is the SALSA cohort, b is the PATH cohort, and d is the SLAS2 cohort reported within one paper

Subgroup analyses of studies using a self-report measure of sedentary time suggested that more sedentary time was significantly associated with better cognitive function (Fig. 11; *r* = 0.037 [95% CI − 0.019, 0.054], *p* < 0.001). Heterogeneity between the studies was statistically significant (*Q* = 28.994, *df* = 7, *p* < 0.001) and large in magnitude (*I*^2^ = 76%) (Fig. 12).

#### Subgroup Analyses of Cognitive Domains from Self-Report Studies

**Fig. 13:**
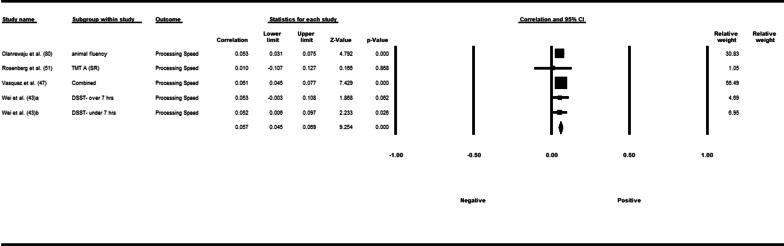
Forest plot of all eligible studies for the meta-analysis that used self-report as the method a measurement for sedentary behaviour reporting on the association of sedentary behaviour with the cognitive domain of processing speed using a random-effects model. *TMT A* Trail Making Test A, *DSST* Digit Symbol Substitution Test. Note a is a population with over 7 h of sleep, and b is a population with under 7 h of sleep

There were sufficient studies to meta-analyse the relationship of self-reported sedentary time with two of the seven domains of cognitive function. Global cognitive function showed no significant association (Fig. 13; *r* = 0.024 [95% CI − 0.005, 0.053], *p* = 0.106; *Q* = 20.827, *df* = 5, *p* < 0.05, *I*^2^ = 76%) while processing speed showed a significant positive association (Fig. 13; *r* = 0.057 [95% CI 0.045, 0.069], *p* < 0.001; *Q* = 1.014, *df* = 4, *p* = 0.908, *I*^2^ = 0%).

#### Prevalence of Sedentary Behaviour and Cognitive Function

**Fig. 14:**
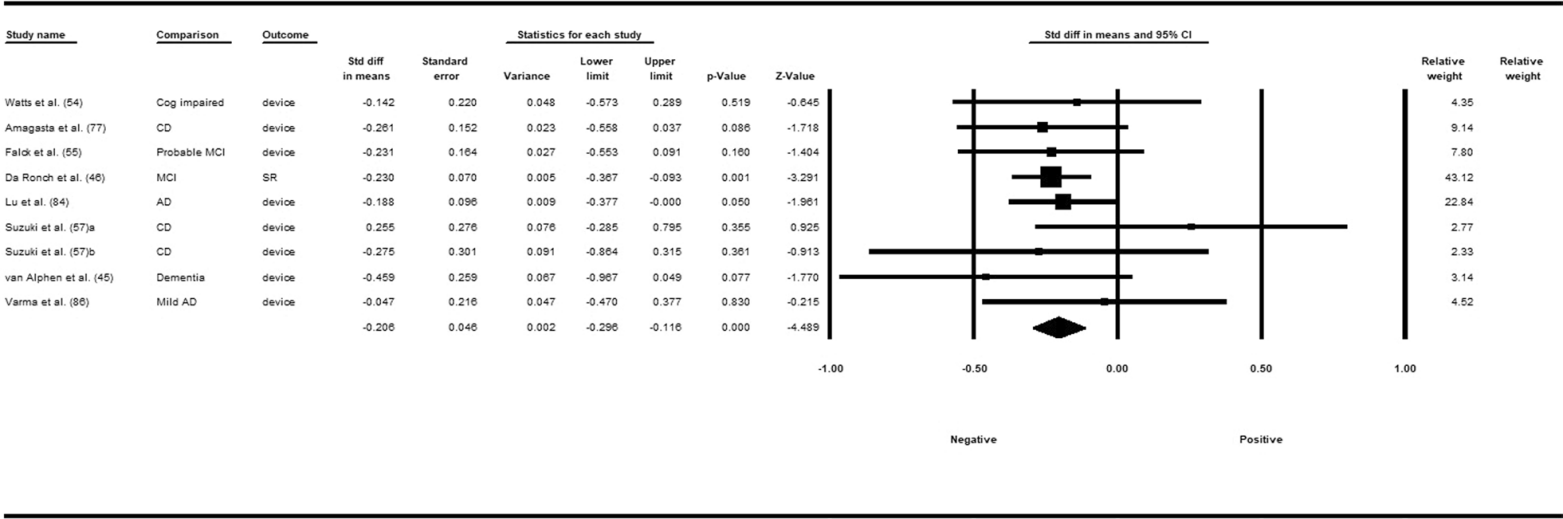
Forest plot of all eligible studies for the meta-analysis that reported on the prevalence of sedentary time for a clinical (i.e., diagnosed with cognitive impairment) versus non-clinical (i.e., cognitively healthy) population. a are females, and b are males. Cog impaired, cognitively impaired, *CD* cognitive decline, *MCI* mild cognitive impairment, *AD* Alzheimer’s disease

Eight studies provided sufficient data to be included in the meta-analysis examining the prevalence of sedentary behaviour in cognitively impaired versus cognitively healthy populations. The random effect model showed a significant standard difference in mean (SDM) hours spent sedentary (Fig. 14; SDM = −0.219 [95% CI − 0.310, − 0.128], *p* < 0.001; *Q* = 1.858, *df* = 7, *p* = 0.967, *I*^2^ = 0%) for cognitively impaired populations spending more time sedentary when compared to cognitively healthy populations.

## Discussion

The purpose of this paper was to systematically review and quantify the size and direction of the relationship of total sedentary time with cognitive function and/or cognitive impairment under selected moderator conditions (i.e., method of sedentary time measurement, the cognitive domain being examined, categorical (i.e., high versus low) sedentary time cut-offs and pattern of sedentary time accumulation). We also aimed to explore the prevalence of sedentary time in populations diagnosed with mild cognitive impairment or dementia versus populations deemed as being cognitively healthy in middle-aged and older adults. Furthermore, we aimed to investigate whether experimental studies aiming to reduce or break up sedentary time affect cognitive function. Lastly, we aimed to conduct a quantitative pooled analysis of all individual studies through meta-analysis procedures to derive conclusions about these relationships. While other reviews have been conducted on the relationship of sedentary behaviour with cognitive function, to our knowledge, this is the first to quantify the association using a meta-analysis. Based on the pooled co-efficient estimates, we found a relationship of sedentary time with cognitive function, but the direction differed depending on the moderator being assessed. More specifically, there was a detrimental relationship for studies using a device to measure sedentary time while studies using self-report tended to find a beneficial relationship. For the device-based studies, more sedentary time was shown to be associated with worse cognitive function when assessing global cognitive function or processing speed. For the self-report-based studies, more sedentary time was shown to be associated with better cognitive function when assessing processing speed. We were unable to perform a meta-analysis on the studies assessing sedentary time using categorical cut-offs, pattern of sedentary time accumulation, or the experimental studies due to large heterogeneity. We were able to perform a meta-analysis for the prevalence studies and found that cognitively impaired populations spend significantly more time sedentary compared to non-impaired populations. Beyond these general findings, the following specific findings warrant commentary.

The variability within the various methods of sedentary time measurement (i.e., self-reported versus device-based) has been highlighted in previous literature [23] and our findings further demonstrate how it continues to be an obstacle. It is important to note that recall bias from self-reporting sedentary time is an issue for all age-groups and populations, but especially in older adults who may be more susceptible to cognitive impairment; as older adults have more memory complaints which may be related to poorer understanding of the questions due to cognitive impairment [87]. This could be a reason for the discrepancy in our findings and is supported by our meta-regression which showed that measurement type (i.e., device versus self-report) was a significant predictor in the overall model (~ 68%). However, this is not to suggest that we should abandon self-report methods, as previous studies have also indicated the importance of self-report measures for capturing the context of behaviour (i.e., television viewing versus reading or writing) [23]. Furthermore, due to the definition of sedentary behaviour being distinct from a lack of physical activity, it is imperative to differentiate between the two when assessing each behaviour. Many of the studies included in the current review that did measure sedentary time with a device were unable to distinguish physical inactivity (i.e., lack of movement) from sedentary time (i.e., low energy expenditure in a seated or lying posture). For example, data from a hip-worn ActiGraph GT3X + is shown to consistently underestimate time spent sitting compared to the thigh worn activPAL™ monitor [54] due to its lack of ability to distinguish sitting from standing still. Future studies need to ensure that they are not reporting physical inactivity (i.e., not meeting the physical activity guidelines) as sedentary time in order to properly advance this field of research. Additionally, future work in this field should perform a meta-analysis on studies only using an activPAL™ and compare the results to studies using other devices when there is more available research. Bias could also be due to different measures of sedentary time (i.e., total sedentary time, percent sedentary in waking hours, 24-h wear period, etc.). However, total sedentary time and percent sedentary during waking hours should be equivalent as sedentary behaviour does not include sleep, and for that reason we excluded studies that reported sedentary behaviour that included sleep time.

We also found the relationship of sedentary time with cognitive function may differ depending on which cognitive domain was being assessed (e.g., working memory, processing speed, etc.). Amongst the various cognitive domains that were assessed, when using a device, more sedentary time was shown to be associated with worse cognitive function when assessing global cognitive function or processing speed. When using a self-report measure of sedentary time, more time spent sedentary was associated with better cognitive performance when assessing processing speed. While our findings highlight variability in the domain of processing speed, it is premature to conclude that it is the most important cognitive domain at this time. However, it is noted as the domain most strongly correlated with impairments in everyday functions [30]. The differences in the associations found for processing speed between device-based and self-reported measured sedentary time studies illustrates how important the measure of sedentary time is (as discussed in the previous paragraph), since the same task can show different associations. For example, the Digit Symbol Substitution task showed a negative association in the device-based forest plot (Fig. 7) and a positive association in the self-report forest plot (Fig. 13). These findings are important as certain parts of the brain may be affected by sedentary behaviour more so than others. For example, one study found that sedentary behaviour is negatively associated with white matter volume, but not grey matter volume [88]. Additionally, cognitive processing speed has been closely related to the structural integrity of white matter [89]. We hypothesize that high levels of sedentary behaviour might be affecting regions of the brain involved in specific domains (i.e., processing speed), more so than others (i.e., working memory). While the specific regions of the brain involved in such tasks are beyond the scope of this review, we believe it to be an important point to bring up for future research to consider. Compared to a previous review on sedentary behaviour and cognitive function, our meta-analysis findings are only consistent for global cognitive function. Falck et al. (2016) indicated associations with memory, executive function and global cognitive functioning. The numerous measures used to assess cognition, even when assessing the same domain is problematic as it makes it challenging to compare studies [24]. For example, Bojsen-Moller et al. (2019) use two different tests to assess processing speed (Trail Making Test A and Digit Symbol) and four different tests to assess working memory (Digit Span Backwards, N-Back, Automated Operation Span, Trail Making Test B) [50] while Kojima et al. (2019) use one test for processing speed that Bojsen-Moller et al. (2019) did not use (Symbol Digit Modality Test) and similarly, two different tests to assess working memory (Symbol Trails and Design Memory) [40]. Falck et al. (2016) intended to amend this problem by recommending exact instruments to be used for each domain going forward, however, this still remained an issue in the current review. Without analogous measures going forward, conclusions about how many and which specific domains are affected by this behaviour are not possible. Furthermore, there needs to be agreement throughout the literature as to which specific domain each cognitive task is assessing. For example, one study used the symbol digit modalities test and deemed it as assessing executive attention [90], while another deemed it as a measure of visual/spatial processing speed and working memory [79]. Without consensus moving forward, we will not be able to better understand and advance the understanding of this relationship.

There were not enough homogenous studies to meta-analyse the relationship of sedentary time as a categorical variable (i.e., high versus low) with cognitive function. For example, one study classified ‘high’ sedentary time as eight or more hours per day, comparing it to those with ‘low’ sedentary time (i.e., less than 8 h) [52] whereas another study used tertiles, separating their participants into three groups (< 180 min/day, > 180 < 308.61 min/day, > 308.61 min/day) [82]. Future studies need to explore if a dose–response relationship exists, not just investigate linear relationships. This is supported by the findings of our meta-regression, in that sedentary time (in hours) was shown to have a significant moderating effect on the overall model. However, there are currently no universally set cut points, as each author determines the cut points for each independent study. Therefore, exploring sedentary time and its association with cognitive function should be investigated using both continuous and categorical variables to investigate any underlying relationship or threshold cut-off.

The current study was not able to provide substantial insight on how the association of sedentary time with cognitive function may differ based on the pattern of accumulation. However, previous studies have shown that there is a difference between long-uninterrupted bouts of sedentary time versus short bouts throughout the day for various health outcomes such as postprandial glucose and insulin responses [91, 92], the low quality and lack of evidence inhibited the ability for us to observe any association. Therefore, more studies are needed before any inferences can be made about how the pattern of sedentary behaviour accumulation is associated with cognitive function.

Interestingly, when looking at the studies reporting on the prevalence of sedentary time for impaired versus healthy populations, we observed a consistent difference. We found that more cognitively impaired people spend significantly more time sedentary compared to their control counterparts. This has ramifications for populations in assisted living environments, as reducing sitting may be a way to reduce or mitigate cognitive decline.

Only eight experimental studies were able to be included in the current review. Due to the large heterogeneity in the populations and designs, results were inconclusive, and we were not able to perform a meta-analysis. Four studies used single day protocol [85, 86, 90, 91] one study used a four-day protocol [58], and three studies used a longer study design (i.e., 8 weeks to 12 months) [79–81]. Of those, two of the longer studies [79, 80] and one of the single day studies [91] showed positive effects of reducing sedentary behaviour on cognitive function. More short and long-term experimental research is needed to explore how reducing or breaking up sedentary behaviour affects cognitive function. Future research should also investigate whether increasing sedentary behaviour (i.e., bed rest studies) affects cognitive function.

Cross-sectionally, our device-based findings are consistent with the previous review by Falck et al. (2016) that suggested higher levels of sedentary time were associated with lower cognitive performance in adults 40 years and over [24]. Although it is difficult to make direct comparisons as this review consisted of mostly studies assessing the relationship of television viewing time as ‘sedentary behaviour’ with cognitive function. Our findings also support the previous review by Copeland and colleagues (2017) in adult 60 years and older in which only half of the studies reported finding associations between increased sedentary time and decreased cognitive function [23]. The findings of our overall meta-analysis can relate to this as without examining the moderators of sedentary time measurement or specific cognitive domains, we would be left with mixed associations. We were able to build upon these previous reviews by differentiating according to the exposure and outcome measures used (i.e., self-report versus device or specific cognitive domains). Similarly, reviews by Loprinzi (2019) [25] and Olanrewaju and colleagues (2020) [26] observed conflicting associations of sedentary time with cognitive function, differing based on sedentary behaviour type (i.e., television viewing, computer use, etc.). This again supports our review and our findings in highlighting how specific moderators vary the direction and strength of the association of sedentary time with cognitive function. It is evident that this field of research is growing at a rapid rate, as 60% of studies included in the current review were published in the year 2017 or later. The heterogeneity in the previous reviews may be the result of differing exposure variables. For example, sedentary behaviour in the current review consisted of ‘total sitting time’, so there is no way to know what specific activities were taking pace during its time; while studies within the aforementioned reviews included domain specific sedentary behaviours (i.e., television watching or computer time only). Although previous research has illustrated that there may be a difference between cognitively demanding sedentary activities (i.e., puzzles) versus passive sedentary activities (i.e., television viewing) [93–95]; we believed the first step was to investigate whether sitting, irrespective of domain, was associated with worse cognitive function, which is why only studies reporting ‘total sedentary time’ were included in the current review. Now that there is an established association of sedentary time and cognitive function, the next step would be to investigate specific domains (i.e., leisure time) or specific sedentary behaviours (i.e., television viewing) to investigate how the association may differ.

There are some important limitations to consider with the current review. First, there were many studies that relied solely on self-reported data which as stated earlier, could impose recall bias. Second, the majority of studies were secondary analyses of a study designed to test a different primary hypothesis. In other words, many studies included in the present review did not have cognitive function as their primary outcome, and thus, may have been underpowered to detect changes in cognitive function. Third, our inclusion criterion for middle-aged and older adults was a mean age of 40 years and older. Therefore, studies could have included participants under 40 years of age, which may reduce the strength of the results. Fourth, the heterogeneity found in the review, both statistically generated and through the extraction of study characteristics. Fifth, the use of studies only published in English. Sixth, bed-rest studies, or studies aiming to increase sedentary behaviour were not included in the systematic review. Future studies should investigate whether reduced sedentary time versus forced extended sedentary time impacts the results. Seventh, we only included high quality studies in the meta-analysis and only performed sub-group analyses when five or more studies were available. Eighth, the bulk of the evidence was from cross-sectional studies. Thus, causality cannot be inferred, and reverse causality remains a possibility. Future studies should aim to investigate the association of sedentary behaviour and cognitive function over different stages of the lifespan. It is important to note that these variables may not show accurate associations from one static point in time. As we age, people sit more, while cognitive function declines [96]. Due to these variables naturally going in opposite directions, it may be more appropriate to use evidence from longitudinal studies assessing change over time. Seventh and lastly, we were not able to examine the association while considering other important factors such as physical activity time or sleep. For example, one study investigated the association of sedentary behaviour and cognitive function with participants who had greater or less than 7 h of sleep and found that sleep did in fact have an impact on the association [43]. That said, while these factors are beyond the scope of the current review, we do believe these are important considerations for future research. The main strength of the current review is that by systematically identifying these various moderators and limitations, future research can be improved. Another strength is the use of large population-based datasets from a range of countries and the subsequent methods used to analyze the data. However, there were limited studies from low-income countries or data on ethnicity. Lastly, by systematically comparing self-report versus device-based studies we were able to identify how the various measurement methods affect the relationship and give recommendations for future research in order to improve upon this.

This systematic review and meta-analysis highlights the need for future studies to use standardized measures of sedentary time and cognitive function. The appropriate device needs to be used to ensure we are capturing sedentary behaviour as opposed to physical inactivity. Furthermore, in order to better understand the association of sedentary time with cognitive function, future research needs to establish categories or cut points of sedentary time that represent ‘high’, ‘medium’ and ‘low’ levels of sedentary time. More studies are needed to investigate change in sitting behaviour over time and how this relates to cognitive function. It is unknown whether reducing sedentary time affects cognitive function in an acute or more long-term nature, so future longitudinal and experimental studies are needed to provide confidence in the findings. There are key transition periods throughout one’s life that can drastically affect sedentary behaviour time. For example, there is evidence showing that physical activity levels tend to decrease while sedentary time increases when transitioning into retirement [97]. Assessing change over time for various populations will allow for a better understanding of the relationship. Lastly, total sedentary time is just one piece of the puzzle, and more work is needed to investigate whether certain domains of sedentary behaviour (e.g.., television viewing, reading, etc.) changes the direction of the association. More research is needed to establish how the relationship of sedentary behaviour with cognitive function may differ when assessing cognitively stimulating sedentary activities (i.e., crossword puzzles) in comparison to non-stimulating domains of sitting (i.e., television viewing). It is hypothesized that the former domains will likely be unrelated to poor cognitive function whereas the latter domains will likely be related to poor cognitive function. Determining the domains of sitting and what is happening while people are sitting, is perhaps the most important consideration for future research.

## Conclusion

In conclusion, within the high heterogeneity of the studies reviewed, our findings suggest that the association of total sedentary time with cognitive function is weak and varies based on the method of sedentary behaviour measurement and cognitive domain being assessed. Specifically, there was a negative association when using a device for global cognitive function and processing speed and a positive association when using self-report for processing speed. Furthermore, our findings suggest that cognitively impaired populations (i.e., diagnosed with mild cognitive impairment or dementia) spend more time sedentary than non-impaired (i.e., cognitively healthy) populations. Overall, these findings suggest that now that we have established that total sedentary time is associated with cognitive function, future research needs to explore how the sedentary time domain (i.e., occupational, leisure, transportation, etc.) and cognitive load associated with each domain (i.e., cognitively stimulating tasks versus non stimulating) may be affecting the association.

## Supplementary Information

**Additional file 1:** Sample Search Strategy.

**Additional file 2:** The definition and acceptable cognitive tests for eachdomain.

**Additional file 3:** Detailed reasons of exclusion.

## Data Availability

Data are available on request from the authors.
