# Supplementary material for: Total Sedentary Time and Cognitive Function in Middle-Aged and Older Adults: A Systematic Review and Meta-analysis
Source: Sports Med Open. 2022 Oct 12;8:127. doi: 10.1186/s40798-022-00507-x (PMC9556686; doi:10.1186/s40798-022-00507-x)
Supplement: Supplementary file 1 — Additional file 1: Sample Search Strategy. [file 40798_2022_507_MOESM1_ESM.docx]

### **Article title:** Sedentary time and cognitive function in middle-aged and older adults: a systematic review and meta-analysis

**Journal:** Sports Medicine  **Authors:** Kirsten Dillon, Anisa Morava, Harry Prapavessis, Lily Grigsby-Duffy, Adam Novic, Paul A Gardiner **Contact:** Kirsten Dillon, Faculty of Health Sciences, The University of Western Ontario, London, Ontario, Canada N6A 3K7. Email: [kdillon9@uwo.ca](mailto:kdillon9@uwo.ca)

### **Supplementary File 1**

### Example search strategy

Below is an example of the search strategy for Scopus:

TITLE-ABS-KEY ( "aging"  OR  "aged"  OR  "middle aged"  OR  elderly  OR  senior  OR  seniors  OR  "older adult"  OR  "older adults"  OR  elderly  OR  geriatric  OR  geriatrics  OR  "old people"  OR  "older-age"  OR  "old age"  OR  adult  OR  adults  OR  "older people"  OR  elders )

TITLE-ABS-KEY ( dementia  OR  intelligence  OR  memory  OR  "Alzheimer Disease"  OR  "Spatial Processing"  OR  "Psychomotor Performance"  OR  attention  OR  "mental deterioration"  OR  "mental decline"  OR  "cognitive deterioration"  OR  "cognitive impairment"  OR  "cognitively impaired"  OR  "cognitive deficit"  OR  "cognitive dysfunction"  OR  cognition  OR  cognitive  OR  dementia  OR  "alzheimer's disease"  OR  "brain function"  OR  "executive function"  OR  "processing speed"  OR  memory  OR  "psychomotor speed"  OR  sensorimotor  OR  "Visual-spatial"  OR  visuospatial  OR  attention  OR  language  OR  intellect )

TITLE-ABS-KEY ( "sedentary behavior"  OR  "sedentary behaviour"  OR  "sedentary lifestyle"  OR  "prolonged sitting"  OR  "sitting time"  OR  "lying time"  OR  "lying down"  OR  "office work"  OR  "sedentary lifestyle"  OR  “sitting  AND time” )
