# Supplementary material for: Total Sedentary Time and Cognitive Function in Middle-Aged and Older Adults: A Systematic Review and Meta-analysis
Source: Sports Med Open. 2022 Oct 12;8:127. doi: 10.1186/s40798-022-00507-x (PMC9556686; doi:10.1186/s40798-022-00507-x)
Supplement: Supplementary file 2 — Additional file 2: The definition and acceptable cognitive tests for eachdomain. [file 40798_2022_507_MOESM2_ESM.docx]

**Article title:** Sedentary time and cognitive function in middle-aged and older adults: a systematic review and meta-analysis

**Journal:** Sports Medicine  **Authors:** Kirsten Dillon, Anisa Morava, Harry Prapavessis, Lily Grigsby-Duffy, Adam Novic, Paul A Gardiner **Contact:** Kirsten Dillon, Faculty of Health Sciences, The University of Western Ontario, London, Ontario, Canada N6A 3K7. Email: [kdillon9@uwo.ca](mailto:kdillon9@uwo.ca)

**Supplementary File 2**

| Domain | Definition | Cognitive tests |
| --- | --- | --- |
| Cognitive flexibility | According to Diamond (2013), “Cognitive flexibility is a component of executive function that requires being able to change perspectives spatially or interpersonally. It builds off the domains of inhibitory control and working memory. It also involves being flexible enough to adjust to changed demands or priorities, to admit you were wrong, and to take advantage of sudden, unexpected opportunities.” (Diamond, 2013) | Letter number sequencing  Symbol cancellation  Symbol Trials  Task Switching  Task switching paradigm  The dimensional change card sort test  Trial making test B  Wisconsin card sorting task |
| Episodic memory | According to Harvey (2019), “This component of the memory system interacts with working memory storage processes to encode, maintain, and retrieve information into and out of longer-term storage. Memory information can be from all sensory types and can also be verbal or nonverbal.”(Harvey, 2019) | Brief Spanish English Verbal learning Test  Brief Visuospatial Memory Test- Revised  California Verbal Learning Test  California Verbal Learning Test- II  Face naming score  Faces subtest from the Wechsler Memory Scale-Third Edition  Free Recall  Hopkins Verbal Learning Test - Revised  Immediate and delayed recall  Logical memory delayed  Logical memory immediate  Logical memory recall  Picture sequence memory test  Selective reminding scores  Verbal memory  Verbal memory Z score  Verbal paired association  Wechsler Memory Scale- revised  Word Recognition |
| Executive Function | According to Harvey (2019), “This cognitive domain is referred to commonly as reasoning and problem solving. The global concept of executive functioning is the set of processes that manifest control over other component cognitive abilities, such that cognitive resources can be effectively utilized to solve problems efficiently and plan for the future. Thus, tasks of problem solving, planning, manipulating mazes, and other complex tasks where management of multiple cognitive abilities are required, fall under the domain of executive functioning.” (Harvey, 2019) | Groton maze learning test  Attention network test  Attentional performance battery  Colour word inhibition  Executive function score from the following: trail making test, verbal fluency from the Delis Kaplan Executive Function System, digit span backwards and mental arithmetic from Wecshler Adult Intelligence Scale, Wisconsin Card Sorting Test.  Flanker or Erikson flanker test  Flexibility Score from the Computer-based Test of Attentional Performance  Mazes  Stroop  Symbol cancellation |
| Global Cognitive Function | As defined by Riello et al. (2021), “*cognitive screening tools* that are typically used at a population screening stage to detect potential cognitive impairment that may raise the suspicion of dementia.” (Riello et al., 2021) | 3MS  Addenbrooke’s Cognitive Examination  Addenbrooke’s Cognitive Examination III  Alzheimer’s Disease Assessment Scale-Cognitive plus  Chinese version of the Ascertain Dementia 8-item questionnaire  Cognitive function overall score  Combined score from: Digit Symbol Coding, Hopkins Verbal Learning Test, n-back, Task switching paradigm)  g-factor test battery: Mini-Mental State Examination, Stroop, letter-digit substitution task, Verbal Fluency 15-word learning test, Purdue pegboard test)  General cognitive ability factor (g) computed from 6 tests taken from the Wecshler Adult Intelligence Scale (Matrix Reasoning, Block Design, Letter-Number Sequencing, Symbol Search, Digit Span Backwards, and Digit Symbol), Moray Houst Test No. 12, Alice Heim 4 test)  Global composite: Digit Symbol Coding, Hopkins Verbal Learning Test, n-back, Task switching paradigm  Hong Kong version of Montreal Cognitive Assessment  Items from Montreal Cognitive Assessment  Mini-Mental State Examination  Modified Mini-Mental State Examination  Montreal Cognitive Assessment  Recommendations of the National Institute on Aging- Alzheimer’s Association  Telephone assessment and interview for Cognitive Status (orientation, serial subtraction, word recall, semantics, sentence repetition, linguistic skills, and attention) |
| Motor Skills and Construction | According to Harvey (2019), “This includes several different basic elements of motor activity. It includes fine motor abilities, including manual dexterity and motor speed, as well as reaction time, and more global skills such as balance.” (Harvey, 2019) | Dear- Leiwald Reaction task  Grooved Pegboard Test  Reaction Test  Reaction time from the Computer- based Test of Attentional Performance  Rey Complex Figure  Simple Reaction Time |
| Processing speed | According to Harvey (2019), “Processing speed refers to cognitive processing assessments that require rapid performance of tasks that range from very simple to complex. The critical feature of processing speed tasks is that participants are instructed from the outset to solve the task as rapidly as possible. Scoring is often in terms of elapsed time or number of correct responses. These tasks are intentionally simple but may have some learning requirements (matching symbols with digits) or executively-oriented performance demands (alternation between letters and numbers).” (Harvey, 2019) | Animal fluency  Block Design  Cognitrone test  Detection test  Digit Symbol  Digit symbol Coding  Digit symbol substitution task  Digits forward  Four-choice RT  Identification test  Letter comparison  Letter fluency  Paced auditory serial addition test  Paced Auditory Serial Addition Test  Pattern comparison  Semantic fluency  Stroop 1  Symbol digit modalities test  Trail making test A  Vegetable fluency  Word fluency |
| Working Memory | According to Harvey (2019), “This is the ability to hold information in consciousness for adaptive use. This can include information from all sensory modalities and includes verbal and nonverbal information. Further, working memory is conceptualized to include two separable components: maintenance of information and manipulation of information.” (Harvey, 2019) | Automated operation span  Colour word inhibition  Computer based task  Design memory  Digit span backwards  Digits backwards  Letter number sequencing  List sorting  N-back  Symbol trials  Trail making test B |

References:

Diamond A. (2013). Executive functions. *Annual review of psychology*, *64*, 135–168. https://doi.org/10.1146/annurev-psych-113011-143750

Harvey, P.D., 2019. Domains of cognition and their assessment. Dialogues Clin. Neurosci. 21, 227–237. <https://doi.org/10.31887/DCNS.2019.21.3/pharvey>

Riello, M., Rusconi, E., & Treccani, B. (2021). The Role of Brief Global Cognitive Tests and Neuropsychological Expertise in the Detection and Differential Diagnosis of Dementia. *Frontiers in aging neuroscience*, *13*, 648310. https://doi.org/10.3389/fnagi.2021.648310
