# Supplementary material for: Total Sedentary Time and Cognitive Function in Middle-Aged and Older Adults: A Systematic Review and Meta-analysis
Source: Sports Med Open. 2022 Oct 12;8:127. doi: 10.1186/s40798-022-00507-x (PMC9556686; doi:10.1186/s40798-022-00507-x)
Supplement: Supplementary file 3 — Additional file 3: Detailed reasons of exclusion. [file 40798_2022_507_MOESM3_ESM.docx]

### **Article title:** Sedentary time and cognitive function in middle-aged and older adults: a systematic review and meta-analysis

**Journal:** Sports Medicine  **Authors:** Kirsten Dillon, Anisa Morava, Harry Prapavessis, Lily Grigsby-Duffy, Adam Novic, Paul A Gardiner **Contact:** Kirsten Dillon, Faculty of Health Sciences, The University of Western Ontario, London, Ontario, Canada N6A 3K7. Email: [kdillon9@uwo.ca](mailto:kdillon9@uwo.ca)

### **Supplementary File 3**

| **Author (year)** | **Title** | **Reason for exclusion** |
| --- | --- | --- |
| Abel, B.; Pomiersky, R.; Werner, C.; Lacroix, A.; Schaufele, M.; Hauer, K.  (2019) | Day-to-day variability of multiple sensor-based physical activity parameters in older persons with dementia | Other- doesn’t report association data |
| Álvarez-Gallardo, I.C.; Estévez-López, F.; Torres-Aguilar, X.C.; Segura-Jiménez, V.; Borges-Cosic, M.; Soriano-Maldonado, A.; Camiletti-Moirón, D.; García-Rodríguez, I.C.; Munguía-Izquierdo, D.; Sierras-Robles, Á.; Delgado-Fernández, M.; Girela-Rejón, M.J.  (2019) | Physical activity, sedentary behaviour, physical fitness, and cognitive performance in women with fibromyalgia who engage in reproductive and productive work: the al-Ándalus project | Exposure |
| Arnardottir, N. Y.; Koster, A.; Van Domelen, D. R.; Brychta, R. J.; Caserotti, P.; Eiriksdottir, G.; Sverrisdottir, J. E.; Sigurdsson, S.; Johannsson, E.; Chen, K. Y.; Gudnason, V.; Harris, T. B.; Launer, L. J.; Sveinsson, T.  (2016) | Association of change in brain structure to objectively measured physical activity and sedentary behavior in older adults: Age, Gene/Environment Susceptibility-Reykjavik Study | Outcome |
| Bakrania, K.; Edwardson, C. L.; Khunti, K.; Bandelow, S.; Davies, M. J.; Yates, T.  (2018) | Associations Between Sedentary Behaviors and Cognitive Function: Cross-Sectional and Prospective Findings From the UK Biobank | Exposure |
| Bantoft, C.; Summers, M. J.; Tranent, P. J.; Palmer, M. A.; Cooley, P. D.; Pedersen, S. J.  (2016) | Effect of Standing or Walking at a Workstation on Cognitive Function: A Randomized Counterbalanced Trial | Population |
| Belala, N.; Schwenk, M.; Becker, C.  (2019) | Quantification and Analysis of sedentary Behavior of cognitively impaired Patients in the geriatric Acute Ward | Other- full text not found |
| Bergouignan, A.; Legget, K. T.; De Jong, N.; Kealey, E.; Nikolovski, J.; Groppel, J. L.; Jordan, C.; O'Day, R.; Hill, J. O.; Bessesen, D. H.  (2016) | Effect of frequent interruptions of prolonged sitting on self-perceived levels of energy, mood, food cravings and cognitive function | Population |
| Bronas, U. G.; Steffen, A.; Dion, C.; Boots, E. A.; Arfanakis, K.; Marquez, D. X.; Lamar, M. (2019) | Sedentary Time and White Matter Hyperintensity Volume in Older Adults | Outcome |
| Carter, S. E.; Draijer, R.; Thompson, A.; Thijssen, D. H. J.; Hopkins, N. D.  (2020) | Relationship Between Sedentary Behavior and Physical Activity at Work and Cognition and Mood | Population |
| Charlett, O.P.; Morari, V.; Bailey, D.P.  (2020) | Impaired postprandial glucose and no improvement in other cardiometabolic responses or cognitive function by breaking up sitting with bodyweight resistance exercises: a randomised crossover trial | Population |
| Chrismas, B.C.R.; Taylor, L.; Cherif, A.; Sayegh, S.; Bailey, D.P.  (2019) | Breaking up prolonged sitting with moderate-intensity walking improves attention and executive function in Qatari females | Population |
| Coelho, L.; Hauck, K.; McKenzie, K.; Copeland, J. L.; Kan, I. P.; Gibb, R. L.; Gonzalez, C. L. R.  (2020) | The association between sedentary behavior and cognitive ability in older adults | Exposure |
| Edwards, M. K.; Loprinzi, P. D.  (2017) | The Association Between Sedentary Behavior and Cognitive Function Among Older Adults May Be Attenuated With Adequate Physical Activity | Exposure |
| Edwards, M. K.; Loprinzi, P. D.  (2018) | Effects of a Sedentary Intervention on Cognitive Function | Population |
| Edwards, M. K.; Loprinzi, P. D.  (2017) | Combined associations of sedentary behavior and cardiorespiratory fitness on cognitive function among older adults | Exposure |
| Ehlers, D.K.; Fanning, J.; Sunderlage, A.; Severson, J.; Kramer, A.F.; McAuley, E.  (2020) | Influence of sitting behaviors on sleep disturbance and memory impairment in breast cancer survivors | Exposure |
| Ehmann, P. J.; Brush, C. J.; Olson, R. L.; Bhatt, S. N.; Banu, A. H.; Alderman, B. L.  (2017) | Active Workstations Do Not Impair Executive Function in Young and Middle-Age Adults | Exposure |
| Ellingson, L. D.; Zaman, A.; Stegemoller, E. L.  (2019) | Sedentary Behavior and Quality of Life in Individuals With Parkinson's Disease | Outcome |
| English, C.; Healy, G. N.; Olds, T.; Parfitt, G.; Borkoles, E.; Coates, A.; Kramer, S.; Bernhardt, J.  (2016) | Reducing Sitting Time After Stroke: A Phase II Safety and Feasibility Randomized Controlled Trial | Other- doesn’t report association data |
| Falck, R. S.; Landry, G. J.; Liu-Ambrose, T.  (2016) | Physical Activity and Sedentary Behaviour are Associated with Cognitive Function in Healthy Older Adults But Not Older Adults with Mild Cognitive Impairment: A Cross-Sectional Study | Other- full text not found |
| Fitzsimmons, P. T.; Maher, J. P.; Doerksen, S. E.; Elavsky, S.; Rebar, A. L.; Conroy, D. E.  (2014) | A daily process analysis of physical activity, sedentary behavior, and perceived cognitive abilities | Population |
| Hamer, M., & Stamatakis, E. (2014) | Prospective Study of Sedentary Behavior, Risk of Depression, and Cognitive Impairment. | Exposure |
| Hartman, S.J.; Nelson, S.H.; Myers, E.; Natarajan, L.; Sears, D.D.; Palmer, B.W.; Weiner, L.S.; Parker, B.A.; Patterson, R.E.  (2018) | Randomized controlled trial of increasing physical activity on objectively measured and self-reported cognitive functioning among breast cancer survivors: The memory & motion study | Exposure |
| Huang, Z.; Guo, Y.; Ruan, Y.; Sun, S.; Lin, T.; Ye, J.; Li, J.; He, L.; Wang, S.; Shi, Y.; Wu, F.  (2020) | Associations of Lifestyle Factors With Cognition in Community-Dwelling Adults Aged 50 and Older: A Longitudinal Cohort Study | Population |
| Kesse-Guyot, E.; Andreeva, V. A.; Lassale, C.; Hercberg, S.; Galan, P.  (2014) | Clustering of Midlife Lifestyle Behaviors and Subsequent Cognitive Function: A Longitudinal Study | Exposure |
| Kesse-Guyot, E.; Charreire, H.; Andreeva, V. A.; Touvier, M.; Hercberg, S.; Galan, P.; Oppert, J. M.  (2012) | Cross-Sectional and Longitudinal Associations of Different Sedentary Behaviors with Cognitive Performance in Older Adults | Exposure |
| Kimura, N.; Aso, Y.; Yabuuchi, K.; Ishibashi, M.; Hori, D.; Sasaki, Y.; Nakamichi, A.; Uesugi, S.; Jikumaru, M.; Sumi, K.; Eguchi, A.; Obara, H.; Kakuma, T.; Matsubara, E.  (2020) | Association of Modifiable Lifestyle Factors With Cortical Amyloid Burden and Cerebral Glucose Metabolism in Older Adults With Mild Cognitive Impairment | Exposure |
| Koyanagi, A.; Stubbs, B.; Vancampfort, D.  (2018) | Correlates of sedentary behavior in the general population: A cross-sectional study using nationally representative data from six low- and middle-income countries | Other- same data as an included study |
| Kriegeskorte, V.  (2014) | Sedentary Lifestyle in old Age Risk of Depression and cognitive Impairment | Other- full text not found |
| Kurita, S.; Doi, T.; Tsutsumimoto, K.; Hotta, R.; Nakakubo, S.; Kim, M.; Shimada, H.  (2019) | Cognitive activity in a sitting position is protectively associated with cognitive impairment among older adults | Exposure |
| Labonte-LeMoyne, E.; Jutras, M. A.; Leger, P. M.; Senecal, S.; Fredette, M.; Begon, M.; Mathieu, M. E.  (2020) | Does Reducing Sedentarity With Standing Desks Hinder Cognitive Performance? | Population |
| Loprinzi, P. D.; Kane, C. J.  (2015) | Exercise and Cognitive Function: A Randomized Controlled Trial Examining Acute Exercise and Free-Living Physical Activity and Sedentary Effects | Population |
| Loprinzi, P. D.; Nooe, A.  (2016) | Executive function influences sedentary behavior: A longitudinal study | Population |
| Maher, J. P. (2019) | Within-day time-varying associations between behavioral cognitions and sedentary behavior in older adults | Other- full text not found |
| Martinez-Sanguinetti, M. A.; Leiva, A. M.; Petermann-Rocha, F.; Troncoso-Pantoja, C.; Villagran, M.; Lanuza-Rilling, F.; Nazar, G.; Poblete-Valderrama, F.; Diaz-Martinez, X.; Celis-Morales, C.  (2019) | Factors associated with cognitive impairment in older adults in Chile | Other- not in English |
| Martinho, K. O.; Dantas, E. H. M.; Longo, G. Z.; Ribeiro, A. Q.; Pereira, E. T.; Franco, F. S.; Goncalves, M. R.; de Morais, K. B. D.; Martins, M. V.; Danesio, J.; Tinoco, A. L. A.  (2013) | Comparison of functional autonomy with associated sociodemographic factors, lifestyle, chronic diseases (CD) and neuropsychiatric factors in elderly patients with or without the metabolic syndrome (MS) | Exposure |
| Matson, T. E.; Anderson, M. L.; Renz, A. D.; Greenwood-Hickman, M. A.; McClure, J. B.; Rosenberg, D. E.  (2019) | Changes in Self-Reported Health and Psychosocial Outcomes in Older Adults Enrolled in Sedentary Behavior Intervention Study | Exposure |
| McArdle, R.; Del Din, S.; Donaghy, P.; Galna, B.; Thomas, A.; Rochester, L.  (2020) | Factors That Influence Habitual Activity in Mild Cognitive Impairment and Dementia | Exposure |
| Moritani, T.; Akamatsu, Y.  (2015) | Effect of Exericse and Nutrition upon Lifestyle-Related Disease and Cognitive Function | Exposure |
| Moyle, W.; Jones, C.; Murfield, J.; Draper, B.; Beattie, E.; Shum, D.; Thalib, L.; O'Dwyer, S.; Mervin, C. M.  (2017) | Levels of physical activity and sleep patterns among older people with dementia living in long-term care facilities: A 24-h snapshot | Other- no association data reported |
| Nemoto, Y.; Sato, S.; Takahashi, M.; Takeda, N.; Matsushita, M.; Kitabatake, Y.; Maruo, K.; Arao, T.  (2018) | The association of single and combined factors of sedentary behavior and physical activity with subjective cognitive complaints among community-dwelling older adults: Cross-sectional study | Exposure |
| Nicola, J. A.; Bettina, B. I.; Emmanuel, S.; Seraina, C.; Medea, I.; Nicole, P. H.  (2020) | Patterns of cross-sectional and predictive physical activity in Swiss adults aged 52+: results from the SAPALDIA cohort | Outcome |
| Peltzer, K.; Phaswana-Mafuya, N.  (2015) | Sitting time and associated factors in older adults in South Africa. | Other- same data set as an included study |
| Pindus, D. M.; Zwilling, C. E.; Jarrett, J. S.; Talukdar, T.; Schwarb, H.; Anderson, E.; Cohen, N. J.; Barbey, A. K.; Kramer, A. F.; Hillman, C. H.  (2020) | Opposing associations between sedentary time and decision-making competence in young adults revealed by functional connectivity in the dorsal attention network | Population |
| Poblete-Valderrama, F.; Rivera, C. F.; Petermann-Rocha, F.; Leiva, A. M.; Martinez-Sanguinetti, M. A.; Troncoso, C.; Mardones, L.; Villagran, M.; Nazar, G.; Ulloa, N.; Martorell, M.; Diaz-Martinez, X.; Lanuza, F.; Garrido-Mendez, A.; Celis-Morales, C.; Representacion Grp Invest, Elhoc  (2019) | Physical activity and sedentary behaviours are associated with cognitive impairment in Chilean older adults | Other – not in English |
| Roman, C.; Arnett, P.  (2020) | The Brain-Body Connection in Aging Neurological Populations: Examining the Impact of Exercise and Sedentary Behavior on Brain Structure and Cognitive Functioning in Older Adults with Multiple Sclerosis | Other- full text not found |
| Rosenberg, D.; Walker, R.; Greenwood-Hickman, M. A.; Bellettiere, J.; Xiang, Y. H.; Richmire, K.; Higgins, M.; Wing, D.; Larson, E. B.; Crane, P. K.; LaCroix, A. Z. (2020) | Device-assessed physical activity and sedentary behavior in a community-based cohort of older adults | Outcome |
| Rostami, M.; Razeghi, M.; Daneshmandi, H.; Hassanzadeh, J.; Choobineh, A.  (2020) | Cognitive and skill performance of individuals at sitting versus standing workstations: a quasi-experimental study | Population |
| Schwartz, B.; Kapellusch, J.M.; Schrempf, A.; Probst, K.; Haller, M.; Baca, A.  (2018) | Effect of alternating postures on cognitive performance for healthy people performing sedentary work | Population |
| Sebastiao, E.  (2020) | Activity behavior and cognitive performance in older adults living in a senior housing facility: the impact of frailty status | Other- doesn’t report association data |
| Simpson, D. B.; Breslin, M.; Cumming, T.; de Zoete, S. A.; Gall, S. L.; Schmidt, M.; English, C.; Callisaya, M. L.  (2021) | Sedentary time and activity behaviors after stroke rehabilitation: Changes in the first 3 months home | Other- full text not available |
| Solis-Urra, P.; Plaza-Diaz, J.; Alvarez-Mercado, A. I.; Rodriguez-Rodriguez, F.; Cristi-Montero, C.; Zavala-Crichton, J. P.; Olivares-Arancibia, J.; Sanchez-Martinez, J.; Abadia-Molina, F.  (2020) | The Mediation Effect of Self-Report Physical Activity Patterns in the Relationship between Educational Level and Cognitive Impairment in Elderly: A Cross-Sectional Analysis of Chilean Health National Survey 2016-2017 | Outcome |
| Sperlich, B.; De Clerck, I.; Zinner, C.; Holmberg, H. C.; Wallmann-Sperlich, B.  (2018) | Prolonged Sitting Interrupted by 6-Min of High-Intensity Exercise: Circulatory, Metabolic, Hormonal, Thermal, Cognitive, and Perceptual Responses | Population |
| Stubbs, B.; Vancampfort, D.; Firth, J.; Schuch, F. B.; Hallgren, M.; Smith, L.; Gardner, B.; Kahl, K. G.; Veronese, N.; Solmi, M.; Carvalho, A. F.; Koyanagi, A.  (2018) | Relationship between sedentary behavior and depression: A mediation analysis of influential factors across the lifespan among 42,469 people in low-and middle-income countries | Exposure |
| Sulzer, P.; Graber, S.; Schaeffer, E.; van Lummel, R.; Berg, D.; Maetzler, W.; Liepelt-Scarfone, I.  (2021) | Cognitive impairment and sedentary behavior predict health-related attrition in a prospective longitudinal Parkinson's disease study | Other- no association data |
| Swank, C. (2011) | Effect of aerobic exercise on cognition and sedentary behavior in persons with Parkinson's disease. | Other- dissertation |
| Vancampfort, D.; Probst, M.; Knapen, J.; Carraro, A.; De Hert, M.  (2012) | Associations between sedentary behaviour and metabolic parameters in patients with schizophrenia | Outcome |
| Vancampfort, D.; Stubbs, B.; Koyanagi, A.  (2017) | Physical chronic conditions, multimorbidity and sedentary behavior amongst middle-aged and older adults in six low- and middle-income countries | Other – same population as an included study |
| Vancampfort, D.; Stubbs, B.; Lara, E.; Vandenbulcke, M.; Swinnen, N.; Koyanagi, A.  (2019) | Correlates of sedentary behavior in middle-aged and old age people with mild cognitive impairment: a multinational study | Other – same population as an included study |
| Vancampfort, D.; Stubbs, B.; Mugisha, J.; Firth, J.; Schuch, F. B.; Koyanagi, A.  (2018) | Correlates of sedentary behavior in 2,375 people with depression from 6 low- and middle-income countries | Outcome |
| Vance, D. E.; Wadley, V. G.; Ball, K. K.; Roenker, D. L.; Rizzo, M.  (2005) | The effects of physical activity and sedentary behavior on cognitive health in older adults | Exposure |
| Vincent, G.E.; Jay, S.M.; Sargent, C.; Kovac, K.; Vandelanotte, C.; Ridgers, N.D.; Ferguson, S.A.  (2018) | The impact of breaking up prolonged sitting on glucose metabolism and cognitive function when sleep is restricted | Population |
| Vivas, A.; Freire, P. C.; Sousa, J. L.; Silva, M. C.; Bortolotti, H.; Costa, E. C.; Elsangedy, H. M.; Fontes, E. B.  (2019) | Relationship Between Fitness and Active-Sedentary Behavior with Cognitive and Emotional Recognition in Elderly: Core Study | Other – conference abstract |
| Wallmann-Sperlich, B.; Bucksch, J.; Schneider, S.; Froboese, I.  (2014) | Socio-demographic, behavioural and cognitive correlates of work-related sitting time in German men and women | Exposure |
| Watts, A.; Walters, R. W.; Hoffman, L.; Templin, J.  (2016) | Intra-Individual Variability of Physical Activity in Older Adults With and Without Mild Alzheimer's Disease | Outcome |
| Waugh, M. D.; Dhanda, R.; Calmbach, W.; Hazuda, H.; Mouton, C. P.  (2002) | Cognitive impairment and leisure time physical activity: Is dementia associated with sedentary behavior in older adults? | Other- full text not found |
| Wei, J. K.; Xie, L. Y.; Song, S. H.; Wang, T. S.; Li, C. W.  (2019) | Isotemporal substitution modeling on sedentary behaviors and physical activity with depressive symptoms among older adults in the US: The national health and nutrition examination survey, 2007-2016 | Outcome |
| Wennberg, P.; Boraxbekk, C. J.; Wheeler, M.; Howard, B.; Dempsey, P. C.; Lambert, G.; Eikelis, N.; Larsen, R.; Sethi, P.; Occleston, J.; Hernestal-Boman, J.; Ellis, K. A.; Owen, N.; Dunstan, D. W.  (2016) | Acute effects of breaking up prolonged sitting on fatigue and cognition: a pilot study | Outcome |
| Zhu, W., Wadley, V. G., Howard, V. J., Hutto, B., Blair, S. N., & Hooker, S. P. (2017) | Objectively Measured Physical Activity and Cognitive Function in Older Adults. | Other- doesn’t report association data |
| Zlatar, Z. Z.; Wierenga, C. E.; Bangen, K. J.; Liu, T. T.; Jak, A. J.  (2014) | Increased Hippocampal Blood Flow in Sedentary Older Adults at Genetic Risk for Alzheimer's Disease | Outcome |
